# A phosphate binding pocket is a key determinant of exo- versus endo-nucleolytic activity in the SNM1 nuclease family

**DOI:** 10.1093/nar/gkab692

**Published:** 2021-08-13

**Authors:** Hannah T Baddock, Joseph A Newman, Yuliana Yosaatmadja, Marcin Bielinski, Christopher J Schofield, Opher Gileadi, Peter J McHugh

**Affiliations:** Department of Oncology, MRC Weatherall Institute of Molecular Medicine, University of Oxford, OX3 9DS, UK; Centre for Medicines Discovery, University of Oxford, ORCRB, OX3 7DQ, UK; Centre for Medicines Discovery, University of Oxford, ORCRB, OX3 7DQ, UK; Chemistry Research Laboratory, University of Oxford, Mansfield Road, Oxford OX1 3TA, UK; Chemistry Research Laboratory, University of Oxford, Mansfield Road, Oxford OX1 3TA, UK; Centre for Medicines Discovery, University of Oxford, ORCRB, OX3 7DQ, UK; Department of Oncology, MRC Weatherall Institute of Molecular Medicine, University of Oxford, OX3 9DS, UK

## Abstract

The SNM1 nucleases which help maintain genome integrity are members of the metallo-β-lactamase (MBL) structural superfamily. Their conserved MBL-β-CASP-fold SNM1 core provides a molecular scaffold forming an active site which coordinates the metal ions required for catalysis. The features that determine SNM1 endo- versus exonuclease activity, and which control substrate selectivity and binding are poorly understood. We describe a structure of SNM1B/Apollo with two nucleotides bound to its active site, resembling the product state of its exonuclease reaction. The structure enables definition of key SNM1B residues that form contacts with DNA and identifies a 5′ phosphate binding pocket, which we demonstrate is important in catalysis and which has a key role in determining endo- versus exonucleolytic activity across the SNM1 family. We probed the capacity of SNM1B to digest past sites of common endogenous DNA lesions and find that base modifications planar to the nucleobase can be accommodated due to the open architecture of the active site, but lesions axial to the plane of the nucleobase are not well tolerated due to constriction around the altered base. We propose that SNM1B/Apollo might employ its activity to help remove common oxidative lesions from telomeres.

## INTRODUCTION

The maintenance of genomic integrity is vital for normal cellular processing. Unrepaired, or mis-repaired, DNA lesions act as impediments to DNA and RNA polymerases preventing the faithful replication and expression of genetic information. Accumulation of DNA lesions is implicated in the aetiology of numerous pathologies and states of cellular degeneration; including cancer (1), neurodegenerative disorders (2) and ageing (3). As such, cells have evolved a robust and complex, DNA damage response and repair systems in which nucleases have essential roles.

Human SNM1B (SNM1B, Apollo, or DCLRE1B) is a 5′-to-3′ exonuclease, which was first identified as one of three vertebrate orthologues of the yeast DNA repair nuclease, *PSO2* (4). SNM1B is essential for normal cellular functioning; SNM1B^–/–^ mice exhibit perinatal lethality (5), and no loss-of-function mutations have been reported in humans. SNM1B appears to contribute to genome stability at two levels. First, SNM1B functions at telomeres where its exonuclease activity is required to facilitate t-loop formation and protect leading strand telomeres from inappropriate activation of non-homologous end-joining (NHEJ)-mediated repair and the formation of chromosome fusions (6–8). Second, SNM1B has been implicated in the repair of DNA lesions, namely, interstrand crosslinks (ICLs) and possibly double-strand breaks (DSBs) (9–11).

SNM1B is a shelterin-associated protein that, under normal cellular conditions, localises to telomeres *via* its interaction with TRF2 (telomeric repeat-binding factor 2) (6–8,12,13). *In vitro* biochemical assays and cellular data indicate that SNM1B resects the 5′-recessed strand at newly replicated leading-strand telomeres, generating the 3′-overhang requisite for t-loop formation (6,7,14–16). Accordingly, loss of SNM1B in mammalian cells results in increased formation of telomere-dysfunction-induced foci (TIFs), increased leading strand telomere chromatid fusions, loss of the 3′-overhang telomeric signal, and induction of senescence (8,15,17). These consequences were not suppressed by the expression of a ‘nuclease-dead’ SNM1B variant, highlighting the essential requirement for SNM1B’s nuclease domain and activity in these processes (14,15). Similarly, if its interaction with TRF2 is disrupted, SNM1B cannot appropriately localise, and a telomere-dysfunction phenotype is observed (18,19).

SNM1B is implicated in DNA damage repair pathways, primarily ICL repair (20,21), but also ionising radiation (IR) induced DSB repair (9). Depletion of SNM1B results in increased sensitivity to DNA crosslinking agents (9–11) and SNM1B interacts with known DNA repair factors: either directly, as with SLX4 (22), MUS81, MRE11 (10); or indirectly, as for FANCD2 (10). There is evidence that SNM1B functions epistatically with FANCD2 (11) and SLX4 (23) in ICL repair. In support of this, isolated, truncated (1–335), recombinant human SNM1B is able to exonucleolytically digest past a site-specific ICL (24).

The SNM1B sequence comprises 532 amino acids, wherein the N-terminal region (amino acids 1–332) contains the catalytic (MBL and β-CASP) domains, and the C-terminal region the protein-protein interaction regions, including the well-characterised TRF2 binding domain (amino acids 498–509) (6–8). SNM1B is a member of the metallo-β-lactamase (MBL) structural superfamily (22), of which the β-CASP (CPSF, Artemis, SNM1, PSO2) nucleic acid processing nucleases are a subfamily. All MBL type enzymes possess a characteristic α/β/β/α MBL core fold that provides a scaffold that supports the active site, which coordinates the metal ions essential for catalysis. The β-CASP domain is inserted within the MBL domain and contains three conserved motifs, which contribute to the active site architecture and catalysis (24,25). Few structures are reported for human β-CASP-MBLs: CPSF-73 (26); SNM1A and SNM1B (24); and SNM1C ((27) and (28)). To date, no crystal structures of any of these with a nucleic acid substrate or relevant analogue have been described.

Here, we present a 1.8 Å resolution structure of SNM1B with two 5′-deoxy-adenosine monophosphate (AMP) molecules in its active site. The two nucleotides are proposed to bind in a similar manner to single-stranded DNA, so enabling us to build a model for substrate binding and experimentally probe the molecular basis of SNM1B’s interaction with DNA, its nuclease activity, and substrate selectivity. We have also examined the structure and function of the breast cancer associated SNM1B variant, H61Y, which is suggested to affect cancer risk based on genome wide association studies (GWAS) (29–32). The H61Y substitution is in the MBL domain and located adjacent to the putative active site (24).

## MATERIALS AND METHODS

### Cloning and expression of human SNM1B

DNA encoding for the core MBL-β-CASP domains of SNM1B (aa 1/2–335) were cloned for expression *via* ligation independent cloning (LIC) into baculoviral expression vectors pFB-CT10HF-LIC (GenBank EF199842), except for the D35A/H36A SNM1B mutant, which was cloned in pFB-LIC-Bse (33). Mutants were generated using site-directed mutagenesis or a megaprimer PCR protocol (34). The transfer vectors containing the SNM1B constructs were transposed into bacmids by recombination in DH10Bac (Life Technologies) for viral infection (33). Baculoviruses were generated by transfection and amplification in *Sf9* insect cells (24). SNM1B proteins were produced in 2 L *Sf9* cells at a density of 2 × 10^6^ cells/ml infected with 6 ml of P2 virus stock and cultures were grown for 72 h at 27°C before being harvested by centrifugation in a JLA8.1 rotor (Beckmann-Coulter) at 900 x g for 30 min. Cell pellets were resuspended in 50 ml cold lysis buffer (50 mM HEPES, pH 7.5, 500 mM NaCl, 10 mM imidazole, 5% glycerol (v/v), 1 mM TCEP, supplemented with protease inhibitors (cOmplete, Mini, EDTA-free Protease Inhibitor Cocktail, Roche)).

### Protein purification

Thawed cell aliquots were lysed by sonication on ice, followed by centrifugation (40 000 g, 40 min). The supernatant was mixed with Ni-Sepharose slurry (prewashed with lysis buffer; GE Healthcare) and incubated, on a rotating mixer at 4°C for 1–2 h. Samples were centrifuged (700 g, 5 min), the supernatant decanted, and the Ni-Sepharose beads were washed twice with 50 ml lysis buffer, resuspended in 30 ml wash buffer (50 mM HEPES, pH 7.5, 300 mM NaCl, 45 mM imidazole, 5% glycerol (v/v), 1 mM TCEP), and added to a 25 ml gravity flow column. The column was washed with 10 column volumes (CV) wash buffer, and eluted in 10 CV elution buffer (50 mM HEPES, pH 7.5, 300 mM NaCl, 500 mM imidazole, 5% glycerol (v/v), 1 mM TCEP). SNM1B containing fractions were pooled, and the respective tags cleaved by incubation with TEV protease (overnight, 4°C), whilst being dialysed using a 3.5 kDa molecular weight cut-off SnakeSkin membrane (ThermoFisher Scientific) into a buffer containing 50 mM HEPES, 500 mM NaCl, 5% glycerol, 1 mM TCEP. Following dialysis, the protein solution was added to a 5 ml Ni-Sepharose column. The column was washed iteratively with buffers (as for dialysis) containing increasing amounts of imidazole (10, 45, 80, 150, 300 mM). SNM1B proteins were further purified by size exclusion chromatography using a 16/60 Superdex S200 column (GE, LifeSciences) in a buffer containing 50 mM HEPES, pH 7.5, 500 mM NaCl, 5% glycerol (v/v), 1 mM TCEP. The presence and purity of each SNM1B protein was validated by SDS-PAGE, and intact mass analysis (ESI-TOF-MS) (Supplementary Figure S1). A ‘nuclease-dead’ D35A/H36A SNM1B was including as a negative control for the purification process, to ensure no contaminating nucleases were present (Supplementary Figure S2). D35A/H36A SNM1B, as well as the single mutant, D35A, have previously been reported as ‘nuclease-dead’ in previous studies (5,35). All purified SNM1B proteins with the C-terminal deca-histidine tag were observed to have a mass differential of +42 Da and this was confirmed to be acetylation of the start methionine by post-translational modification mapping by proteolytic digest with trypsin, chymotrypsin, and pepsin and subsequent tandem MS (MS/MS). Both D275A and T257A also show a +16 Da mass difference, consistent with addition of an oxygen atom.

### Protein crystallisation

Crystallisation of the apo forms of WT and H61Y SNM1B was achieved by sitting drop vapour diffusion, at 4°C, in conditions containing 6% PEG 20 K, 0.1 M bicine, pH 7.3 for WT SNM1B; and 6% PEG 3350 K, 0.1 M HEPES, pH 7.0, 0.2 M sodium formate for H61Y SNM1B. Protein concentrations were 15 and 12 mg/ml for WT and H61Y SNM1B, respectively. For co-crystallisation of WT SNM1B with 2′-deoxy-5′-AMP, WT SNM1B (at 15 mg/ml) was incubated with 50 mM 2′-deoxy-5′-AMP (Sigma Aldrich; adjusted to pH 7.5 with 1 M NaOH) for 30 min on ice. Crystallisation was achieved by sitting drop vapour diffusion at 20°C, in conditions containing: 20% PEG 3350 K, 0.2 M ammonium chloride. Crystals were cryoprotected in the reservoir solution supplemented with 20% ethylene glycol, and plunged into liquid nitrogen.

### Data collection and refinement

Data were collected at Diamond Light Source utilising beamlines I02 (H61Y and WT apo form), and I04 (nucleotide form). Diffraction data were processed using DIALS. The structures of SNM1B (apo form) and H61Y SNM1B were solved by molecular replacement using PHASER with PDB: 5AHO used as a search model. The structure of SNM1B (nucleotide form) was also solved using molecular replacement, using SNM1B (apo form) as a search model. Model building was performed using COOT, and subsequent refinement of the native dataset using REFMAC. Data collection and refinement statistics are in Table 1.

**Table 1.** Data collection and refinement statistics

	WT APO	H61Y	Nucleotide form
Space group	*P* 4_1_ 2_1_ 2	*P* 4_1_ 2_1_ 2	*P* 1
Cell dimensions, *a,b,c* (Å)	90.4, 90.4, 104.2	90.7, 90.7, 104.1	50.1, 54.2, 72.1
Angles α, β, γ (°)	90, 90, 90	90, 90, 90	75.4, 74.3, 63.1
Wavelength (Å)	0.979	0.979	0.979
Resolution (Å)	45.2–2.80 (2.95–2.80)	45.4–3.10 (3.31–3.10)	68.6–1.80 (1.84–1.80)
*R*_merge_	0.18 (2.13)	0.15 (2.17)	0.06 (0.62)
*R*_p.i.m._	0.04 (0.44)	0.05 (0.61)	0.05 (0.52)
I/σI	11.8 (1.6)	13.8 (1.3)	7.9 (1.3)
CC1/2	0.99 (0.67)	0.99 (0.58)	0.99 (0.65)
Completeness (%)	100 (100)	100 (100)	93.3 (95.5)
Multiplicity	25.3 (25.3)	12.7 (13.3)	2.2 (2.3)
No. Unique reflections	11188 (1588)	8347 (1474)	55714 (3368)
**Refinement statistics**			
Resolution	45.2–2.80	45–3.1	44–1.80
*R*_work_/*R*_free_ (%)	26.6/32.1	26.8/31.9	17.7/22.3
No. atoms			
Protein	2447	2449	5200
Solvent	1	1	566
Ligand/ion	1	1	139
Average *B* factors (Å^2^)			
All atoms	80	91	32
Protein	80	91	31
Solvent	-	-	38
Ligand/ion	105	119	49
Wilson *B*	86	103	24
R.M.S. deviations			
Bond lengths (Å)	0.003	0.002	0.007
Bond angles (°)	0.5	0.5	0.9
Ramachandran plot			
Favoured (%)	93.5	93.2	97.7
Allowed (%)	99.35	99.68	100
PDB ID		7B2X	7A1F

*Data in parentheses are for the high-resolution shell.

### Substrate preparation

10 pmol of single-stranded DNA (Eurofins MWG Operon, Germany) were labelled with 3.3 pmol of α-^32^P-dATP (Perkin Elmer) by terminal deoxynucleotidyl transferase (TdT, 20 U; ThermoFisher Scientific), incubated together at 37°C for 1 h. This solution was passed through a P6 Micro Bio-Spin chromatography column (BioRad), and the radiolabelled DNA was annealed with the appropriate unlabelled oligonucleotides (1:1.5 molar ratio of labelled to unlabelled oligonucleotide) (Supplementary Table S1 for sequences) by heating to 95°C for 5 min, and cooling to below 30°C in annealing buffer (10 mM Tris–HCl; pH 7.5, 100 mM NaCl, 0.1 mM EDTA). Annealed substrates were validated by non-denaturing PAGE (Supplementary Figure S3).

### Gel-based nuclease assays

Standard exonuclease assays were carried out in 10 μl final volume reactions containing 20 mM HEPES-KOH, pH 7.5, 50 mM KCl, 10 mM MgCl_2_, 0.05% Triton X-100, 5% glycerol and 1 nM of SNM1B. Reactions were started by the addition of DNA substrate (10 or 100 nM), incubated at 37°C for the indicated time period, and quenched by the addition of 10 μl stop solution (95% formamide, 10 mM EDTA, 0.25% xylene cyanol, 0.25% bromophenol blue) and incubating at 95°C for 3 min.

Reactions were analysed by 20% denaturing polyacrylamide gel electrophoresis (40% solution of 19:1 acrylamide:bis-acrylamide, BioRad) and 7 M urea (Sigma Aldrich)) in 1× TBE (Tris-borate EDTA) buffer. Electrophoresis was carried out at 700 V for 75 min; gels were subsequently fixed for 40 min in a 50% methanol, 10% acetic acid solution, and dried at 80°C for 2 h under a vacuum. Dried gels were exposed to a Kodak phosphorimager screen and scanned using a Typhoon 9500 instrument (GE).

### Real-time kinetic measurements

Real-time fluorescence nuclease assays were carried out using a modified version of that previously described (Supplementary Figure S4) (24,35). Reactions were carried out in black 384-well microplates (Corning, USA), with a total volume of 15 μl. To investigate kinetics, reactions contained between 10 and 2500 nM DNA substrate (Supplementary Table S1), 0.5 nM SNM1B, in the same reaction buffer as for gel-based nuclease assays. The fluorescence spectra were measured using a PHERAstar FSX (excitation: 495 nm; emission: 525 nm) with readings taken every 150 s at 37°C (Supplementary Figure S10). Michaelis–Menten enzyme kinetics were calculated using Prism software (GraphPad Software Inc., La Jolla, CA, USA).

### Fluorescence polarisation DNA binding assays

Reactions (volume 20 μl) were carried out in black 384 well plates (Corning, USA). SNM1B protein was serially diluted from 5 μM to 0.288 nM and incubated with DNA substrate (Supplementary Table S1) at 25 nM final concentrations in a buffer containing 20 mM HEPES, pH 7.5, 50 mM KCl, 0.5 mM TCEP at room temperature for 5 min. Fluorescence polarisation was measured as soon as possible following probe addition with excitation at 285 nm and emission at 520 nm using a PHERAstar FS plate reader (BMG Labtech). Fluorescence polarisation was plotted against log_10_ SNM1B concentration, and binding curves were fitted using a non-linear regression (curve fit) in GraphPad Prism (GraphPad Software Inc., La Jolla, CA, USA).

## RESULTS

### A structure of SNM1B_1–355_ complexed with two 2′-deoxy-5′-adenosine monophosphate molecules at its active site

To gain insight into the SNM1B reaction mechanism, we obtained crystal structures with relevant active site binding ligands, solving a co-crystal structure of a C-terminally truncated form of SNM1B (amino acids 1–335) with two 2′-deoxy-5′-adenosine monophosphate (AMP) molecules at the active site (denoted SNM1B (nucleotide form)). All biochemical and crystallographic experiments were carried out with truncated SNM1B_1–355_ comprising the catalytic MBL and β-CASP domains, as production of the full-length protein is not well tolerated in bacterial or insect cell expression systems. Small amounts of the full-length SNM1B protein have been previously purified by other research groups (6,16), and the full-length protein exhibits 5′–3′ exonuclease activity consistent with that of the truncated SNM1B protein, containing only the N-terminal MBL and β-CASP domains (35).

**Figure 1.**
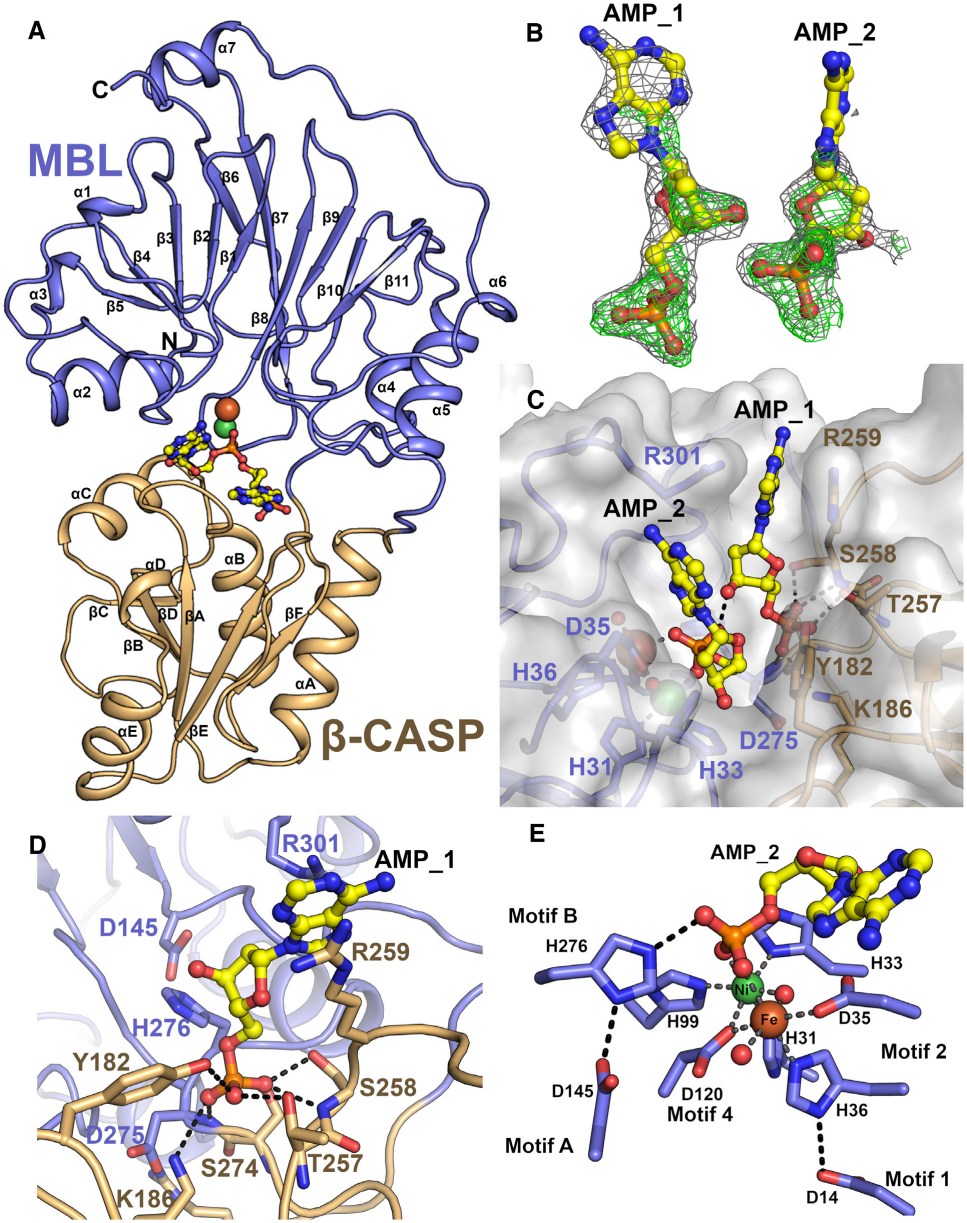
The structure of SNM1B_1–355_ with two 2′-deoxy-5′-AMP molecules at the active site. (**A**) Overall fold of SNM1B_1–355_ co-crystallised with two AMP molecules at the active site. The MBL and β-CASP domains are as indicated, with the MBL domain in blue, and the β-CASP in gold. The β-strands and α-helices of the MBL and β-CASP domains, as well as the N- and C-termini are as labelled. The β-CASP domain is inserted between β-strands 10 and 11 of the MBL domain. (**B**) The electron density map of the two nucleotides bound to the active site of SNM1B_1–355_. The 2*F*_o_– 1*F*_c_ map is shown in light grey (contoured at 1.0 sigma) and the omit *F*_o_– *F*_c_ map is shown in green (contoured at 3.0 sigma). The density of the nucleotides is of good quality with the exception of the adenine base of the second nucleotide which is presumably disordered. See Supplementary Figure S7 for the electron density of the extended active site of SNM1B_1–355_ (nucleotide form). (**C**) Surface view of SNM1B_1–355_ with the two AMP molecules bound, oriented towards the active site. Residues that contribute to binding and/or catalysis are as labelled. The active site metal ions (Ni^2+^ at the M1 site, and Fe at the M2 site) are depicted as spheres. (**D**) The coordination network of the phosphate group of AMP_1 defines the phosphate binding pocket in the active site of SNM1B_1–355_. Contributing hydrogen bonds are depicted as dashed lines. (**E**) The coordination network of the phosphate group of AMP_2 bound to the SNM1B_1–355_ active site reveals residues likely involved in catalysis. Active site metal ions (Ni^2+^ at the M1 site, and Fe at the M2 site, are as indicated). The conserved catalytic motifs from the MBL domain are as indicated.

Similar to the previously reported structure of truncated SNM1B (24), our structure (1.8 Å resolution) exhibits the bilobar MBL and β-CASP domain architecture, characteristic to this sub-family of nucleic acid processing MBLs (26,36–38) (Figure 1A). The overall fold consists of a four-layered β-sandwich (α/β-β/α) with two mixed β-sheets, flanked by two α-helices on each side, with the di-metal binding site located at the edge of the β-sandwich. The β-CASP domain is inserted between β10 and β11 of the MBL domain and consists of a fully parallel β-sheet, flanked by three α-helices on one side, and one α-helix on the other. The catalytic core is interfaced between the two domains, with residues from both domains likely contributing to DNA binding and hence catalysis.

The two 2′-deoxy-5′-AMP (AMP_1 and AMP_2) molecules are bound in the active site and are arranged in a position consistent with that predicted for the terminal nucleotide products from hydrolysis of a ssDNA substrate. The P–P distance is 6.1 Å, commensurate with the distance between two phosphate groups of adjacent nucleotides in ssDNA (∼6.6 Å). The electron density map (Figure 1B, Supplementary Figure S7) reveals the two phosphate groups are well-resolved, with their binding stabilised by specific interactions with residues in the extended active site. The ribose sugars of both nucleotides, and the adenine base of AMP_2 were refined with full occupancy and are well resolved, with the adenine base of AMP_1 being less well resolved in the electron density maps, possibly in part, reflecting flexibility around the glycosidic bond (Figure 1B).

The first nucleotide (AMP_1) is bound in a well-defined pocket formed in the cleft between the MBL and β-CASP domains (Figure 1C). The 5′-phosphate group is located at the base of this pocket, and makes extensive protein interactions, including seven hydrogen bonds *via* the main chain amides of S258 and D275, the sidechain hydroxyls of Y182, T257, S258 and S274, and a single positively charged sidechain of K186 (Figure 1C, D). Whilst the ribose sugar does not appear to form any direct protein interactions, binding of the adenine base is stabilized by stacking with the guanidium sidechains of R301 and R259 (Figure 1D), involving cation-π type interactions.

### Defining the importance of the phosphate binding pocket to SNM1B_1–355_ DNA binding and its nuclease activity

**Figure 2.**
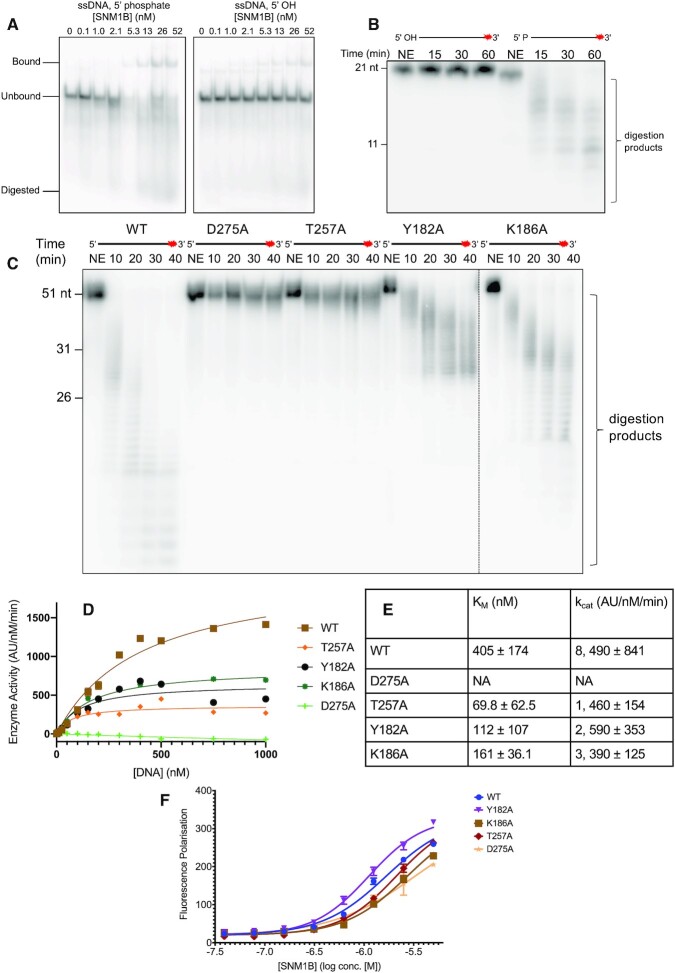
Identifying features that contribute to nucleotide binding in the extended active site of SNM1B _1–355_. (**A**) WT SNM1B_1–355_ preferentially binds ssDNA with a 5′ phosphate, compared with a 5′ hydroxyl moiety. EMSA showing binding of WT SNM1B_1–355_ to 21 nt ssDNA substrate either with a 5′ phosphate group, or a 5′ hydroxyl group. 1 nM of the appropriate DNA substrate was incubated for 5 minutes at 37°C with WT SNM1B_1–355_ (concentrations as labeled), then analysed by 10% native PAGE. Unbound DNA substrate, DNA substrate bound to SNM1B_1–355_, and digestion products are as labeled. (**B**) WT SNM1B_1–355_ will exonucleolytically process ssDNA with a terminal 5′ monophosphate, but not a terminal 5′ hydroxyl group. 0.8 nM SNM1B_1–355_ was incubated with 100 nM ssDNA at 37°C for the indicated time. Reaction products were subsequently analysed by 20% denaturing PAGE. The red asterisk indicates the 3′ radiolabel on the ssDNA substrate. The size of oligonucleotide markers is indicated on the left-hand side of the gel (length in nucleotides). (**C**) Specific residues within the phosphate binding pocket are required for nuclease activity and mutations within this region have varying negative effects on the nuclease activity of SNM1B. 1.0 nM SNM1B_1–355_ was incubated with 10 nM ssDNA at 37°C for the indicated time period. Reaction products were analysed by denaturing PAGE, as in (B). (**D**) Michaelis-Menten enzyme kinetics for WT and phosphate binding pocket mutant SNM1B_1–355_ proteins. Data obtained *via* real-time fluorescence-based nuclease assay, with at least three experimental repeats for each. 0.25 nM SNM1B_1–355_ was incubated with increasing amounts of DNA substrate (between 1.0 and 1000 nM) and fluorescence readings were taken every 150 sec for 35 min. Error bars indicate the SEM. See Supplementary Figure S8 for the raw data plotted with the increase in fluorescence intensity against time for WT SNM1B_1–355_. All data were obtained from at least four experimental repeats. (**E**) Table showing the *K*_M_ and *k*_cat_ values for WT and phosphate binding pocket mutants of SNM1B_1–355_. Values determined by fitting Michaelis-Menten curves using Graphpad-Prism software. The ± error values indicate the 95% confidence interval. (**F**) Substitutions within the phosphate binding pocket do not significantly affect the DNA binding ability to SNM1B_1–355_. DNA binding determined by a fluorescence polarisation DNA binding assay for WT and phosphate binding pocket mutants of SNM1B_1–355_. 50 nM 3′ overhang DNA substrate, containing a 3′ fluorescein (Supplementary Table S1), was incubated with serially diluted SNM1B_1–355_ (concentrations as indicated) for five minutes at room temperature, before fluorescence polarisation was measured. Error bars indicate the SD. All data were obtained from at least three experimental repeats. It is important to note that the binding curves do not saturate, and we did not possess enough SNM1B_1–355_ for each of the substitution mutations to test concentrations higher that 1 μM SNM1B_1–355_. Therefore, absolute *K*_D_ values should be interpreted cautiously, although qualitative changes in binding affinity can be observed from the DNA binding curves. All gels shown are representative of at least three individual experiments, which show qualitatively comparable results.

SNM1B prefers to bind a substrate possessing a terminal 5′-phosphate (Figure 2A) and absolutely requires a terminal 5′-phosphate for catalysis (Figure 2B). This selectivity is likely conferred by extensive interactions with the terminal 5′-phosphate in the 5′-phosphate binding pocket, as defined by our structure (Figure 1D). To probe the requirement of individual residues within this pocket for the nuclease activity and DNA binding of SNM1B_1–355_, we performed an alanine substitution analysis of residues implicated in phosphate binding (D275A, K186A, T257A and Y182A SNM1B (Supplementary Figure S1)). We have been unable to produce the S258A and S274A variants.

Due to the fact that binding in the 5′-phosphate binding pocket involves multiple polar interactions, single alanine substitutions are unlikely to completely abrogate binding; nevertheless, all of the studied substitutions were detrimental to nuclease activity (Figure 2C–E). Both the K186A and Y182A variants exhibited decreased digestion of a ssDNA substrate (the latter in a more pronounced manner than the former). D275A and T257A exhibited negligible digestion of the 51 nt ssDNA substrate with a 40 min reaction (Figure 2C). To investigate the kinetic consequences of these substitutions, a real-time fluorescence-based nuclease activity assay was performed using a 20 nt ssDNA substrate. Mutants K186A, Y182A and T257A, exhibited decreased catalytic turnover, with effects ranging from less severe (K186A) to more severe (T257A), resulting in reduced *k*_cat_ and, (more unexpectedly) *K*_M_ values compared with WT SNM1B. The D275A point substitution completely abrogated nuclease activity (Figure 2D and E). This is perhaps surprising given that this residue interacts with the 5′-phosphate *via* its main chain amide, although it is possible that the D275 sidechain plays a role in the formation of the active site, through charged interactions with the positively charged sidechains of R158 and K186, as well as a hydrogen bond to H99 *via* its main chain carbonyl (Figure 1D).

The effects of these substitutions on DNA binding were investigated, using a 3′ overhang DNA substrate and a fluorescence polarization (FP) assay (Figure 2F). WT SNM1B appears to bind to the probe with an apparent dissociation constant (*K*_D_^app^) in the low micromolar range 1.67 ± 0.17 μM. Most of the phosphate binding pocket variants showed similar or increased apparent dissociation constants (*K*_D_^app^) with K186A and T257A being moderately affected (*K*_D_^app^ 2.73 ± 0.3 and 2.12 ± 0.18 μM respectively); *K*_D_^app^ for Y182A 1.1 ± 0.09 μM, being slightly lower than the wildtype for unclear reasons; and D275A exhibiting the greatest increase in *K*_D_^app^ (3.28 ± 0.4 μM), consistent with this substitution also showing the greatest decrease in nuclease activity. It is important to note, however, that the conditions of the turnover and FP binding assays are different and they use different reporter molecules.

### AMP_2 binds to the SNM1B_1-355_ active site in a manner likely resembling substrate binding

**Figure 3.**
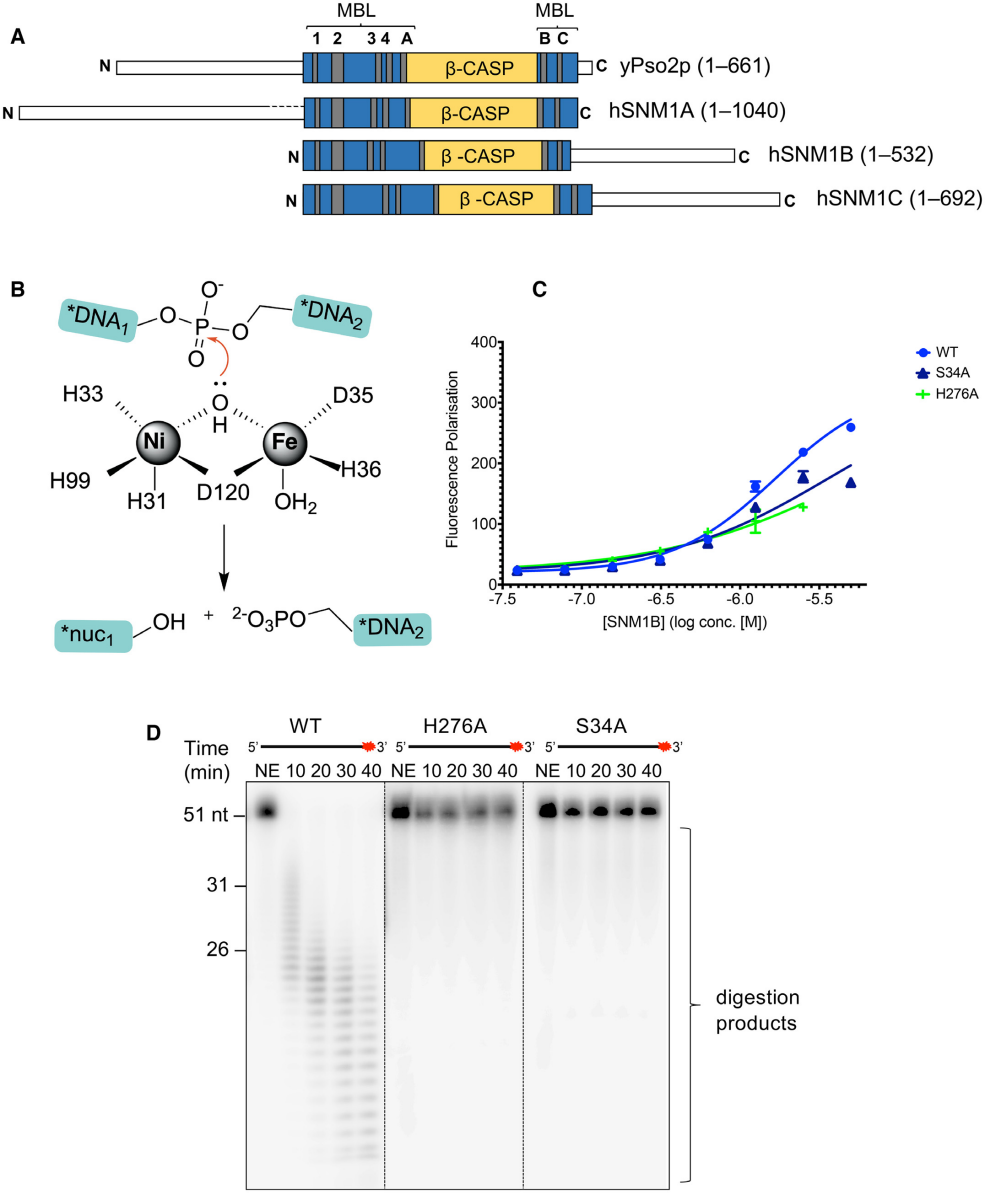
The structure of WT-SNM1B_1–355_ (nucleotide bound) implies catalytic mechanism. (**A**) Alignment of budding yeast Pso2p, human SNM1A, human SNM1B, and human SNM1C sequences, with the conserved motifs in the MBL and β-CASP domains as indicated. (**B**) The proposed reaction mechanism for SNM1B. H31, H33, D35 and H36 constitute motif 2; H99 is motif 3; D120 is motif 4, all from the MBL domain. The product ‘nuc_1_’ in the schematic simply indicates the free nucleotide that is released subsequent to exonucleolytic cleavage. (**C**) H276A and S34A exhibit decreased DNA binding capacity compared with a WT SNM1B_1–355_ on a 3′ overhang duplex DNA substrate. DNA binding determined by a fluorescence polarisation DNA binding assay for WT and phosphate binding pocket mutants of SNM1B_1–355_. 50 nM 3′ overhang DNA substrate was incubated with serially diluted SNM1B_1–355_ (concentrations as indicated) for five minutes at room temperature, before fluorescence polarisation was measured. Error bars indicate the SD, and data were obtained from at least three experimental repeats. Note that as less H276A SNM1B_1–355_ was obtained during the purification process, we were unable to test DNA binding at the highest enzyme concentration compared with S34A and WT SNM1B_1–355_. (**D**) H276 and S34 are required for the nuclease activity of SNM1B_1–355_. H276A and S34A SNM1B_1–355_ exhibit negligible nuclease activity when compared with WT. 1.0 nM SNM1B_1–355_ was incubated with 10 nM ssDNA at 37°C for the indicated time-period. Reaction products were subsequently analysed by 20% denaturing PAGE. The red asterisk indicates the 3′ radiolabel on the ssDNA substrate. The size of oligonucleotide markers is indicated on the left-hand side of the gel (length in nucleotides). Gel shown is representative of at least three individual experiments, which show qualitatively comparable results.

AMP_2 is bound at the SNM1B_1–355_ MBL fold supported active site (motifs 1–4; Figure 3A) which is responsible for metal ion coordination; conserved residues from the β-CASP domain (motifs A–C) also contribute to the active site architecture (Figure 3A). The 5′-phosphate group of AMP_2 is located where the phosphodiester bond is predicted to be, prior to substrate cleavage. Two 5′-phosphate oxygen atoms (O1 and O3) of AMP_2 coordinate to one of the metal ions, whilst the third phosphate oxygen is positioned to form a hydrogen bond / electrostatic interaction with NE2 of imidazole sidechain of H276 (motif B; β-CASP domain) (Figure 1D). Neither the ribose sugar, nor the adenine base make extensive interactions with the protein, although S34 and the acetylated N-terminal methionine make close Van der Waals contacts to the ribose sugar and adenine base, respectively.

The electron density for active site residues of SNM1B**_1–355_**(nucleotide form) indicates both metal ion-binding sites are fully occupied. X-ray fluorescence analysis (XRF) of the crystals revealed that a mixture of nickel and iron were present in roughly equal proportions (Supplementary Figure S5). Although we cannot be sure of the Fe/Ni distribution between the two sites, analysis of related metal ion binding sites led us to model a Ni^2+^ at the M1 site (according to canonical MBL nomenclature), coordinated by H31 and H33 (motif 2; MBL domain), H99 (motif 3; MBL domain), D120 (motif 4; MBL domain), and a water molecule. Octahedral coordination is completed by the 5′-phosphate oxygen (O3) of AMP_2. The second metal ion (M2 site) was modelled as an Fe; it is coordinated in an octahedral arrangement by D35A and H36A (motif 2; MBL domain), D120 (motif 4; MBL domain), an oxygen (O1) of the 5′-phosphate group of AMP_2, and two water molecules (Figure 1E and Supplementary Figure S6). A water or hydroxide (which, in models of the mechanism, is assumed to be activated for nucleolytic attack as a hydroxide ion) bridges the metal ions. This hydroxide is likely to react with the phosphodiester bond, resulting in exonucleolytic cleavage of the terminal nucleotide (Figure 3B). Our placement of Ni and Fe at these sites is provisional but is consistent with the electron density contour levels (approximately 18 and 13 sigma, respectively), and the precedent of previous examples of single metal containing complexes within the family with Ni occupying the first metal site (for example PDB:5AHR [27]).

**Figure 4.**
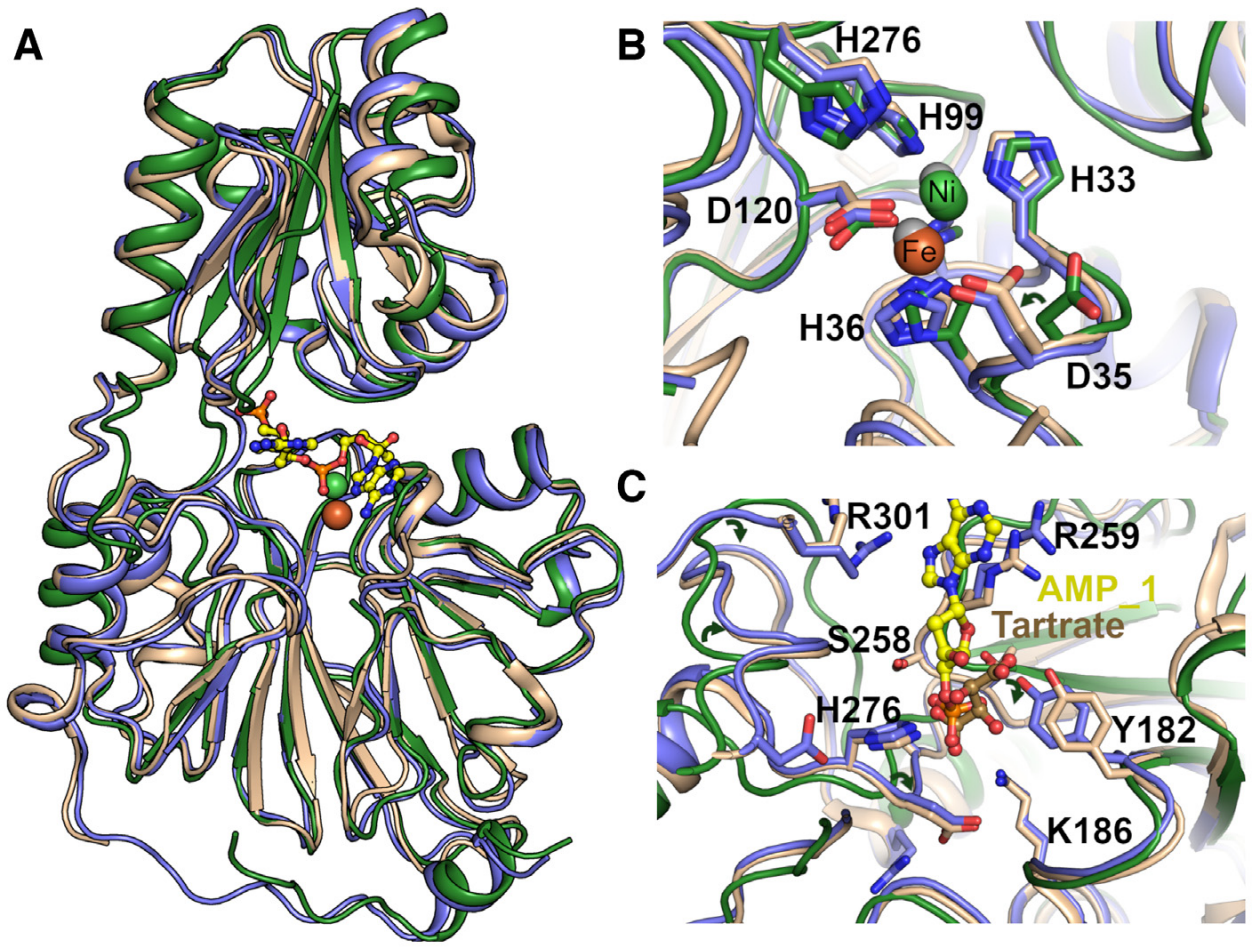
The 5′ phosphate binding pocket of SNM1B_1–355_. (**A**) Overlay of the views from structures unliganded SNM1B_1–355_ (apo form; coloured green), SNM1B_1–355_ (nucleotide form; coloured slate), and SNM1B_1–355_ with a tartrate molecule in the active site (PDB: 5AHO; coloured peach, tartrate not shown). The Ni^2+^ (M1 site) and Fe (M2 site) metal ions present in the nucleotide form are represented as red and green spheres, respectively. The two 2′-deoxy-5′-AMP molecules are represented in stick form. (**B**) Enlargement of the active site of all overlaid SNM1B_1–355_ structures from (A). The metal-ion coordinating residues are as labelled. The side chain orientation of D35 compared between the SNM1B_1–355_ (apo form) and the SNM1B_1–355_ (nucleotide form) is indicated by an arrow. (**C**) Enlargement of the phosphate binding pocket with the three overlaid SNM1B_1–355_ structures (A). The amino acid side chains involved in coordinating the phosphate of AMP_1 from SNM1B_1–355_ (nucleotide form) and the tartrate from SNM1B_1–355_ (PDB: 5AHO) are indicated. The positions of the base stacking residues, R301 and R259, of each are also indicated. SNM1B_1–355_ (nucleotide form) is coloured in slate and SNM1B_1–355_ (PDB: 5AHO) in green.

In addition to the structure of SNM1B_1–355_ (nucleotide form) we solved a structure of unliganded SNM1B_1–355_ to 2.8 Å resolution, hereafter denoted SNM1B_1–355_ (apo form) (Table 1). For the apo form, only the M1 metal binding site was occupied; XRF revealed iron, nickel, and zinc present in the crystal, with nickel being the most abundant (Supplementary Figure S5). This metal ion is coordinated in an approximately octahedral conformation (Supplementary Figure S8), with a bonding network (H31, H33, H99, D120, and a water molecule) and co-ordination distances in the region of 2.3 Å consistent with each Ni^2+^, Zn^2+^, Fe^2+^ (39). D35 which forms part of the second metal ion coordination site (M2) adopts a different sidechain conformation in the apo-structure compared to the nucleotide-form structure (Figure 4B).

Overlays of the structures of SNM1B_1–355_ (apo form) and SNM1B_1–355_ (nucleotide form) (Figure 4A) reveal they have the same architecture with similar relative domain orientations. The most prominent differences lie in the ordering of the extended loop connecting the final β-strand on the MBL domain with the C-terminal helix (residues 297–315), and smaller but still substantial shifts in the two regions connecting the MBL and β-CASP domains (residues 146–159 and 266–279). These regions cluster around the 5′-phosphate binding pocket (Figure 4C), with significant shifts to both main chain and side chains required to accommodate the AMP_1 in the apo form structure, suggesting an induced pocket. The conformation of a reported structure of SNM1B (also 1–355) in complex with tartrate ions (5AHO; (24)) is very similar to the nucleotide bound form described here with the tartrate ion occupying the 5′ phosphate binding pocket and making several of the same contacts formed with AMP_1 (Figure 4C). There are also small changes in the positioning of base stacking residues R259 and R301 which are each disordered in (apo form), but adopt different conformations between the nucleotide and 5AHO structures. Presumably, these regions are relatively flexible and move to form favourable interactions with DNA upon substrate binding.

The presence of each of the ions in both the apo and nucleotide form of SNM1B_1–355_ was confirmed by inductively-coupled-plasma mass-spectrometry (ICP-MS) (Supplementary Figure S9). However, whether these structures represent the catalytically or physiologically relevant forms of SNM1B is not clear, and it is likely that the presence of Ni in both the apo and nucleotide form crystals is due to the use of Ni-Sepharose in the purification process. The role of the metal ions for catalysis is essential, as mutations to the metal ion-binding residues (D35A/H36A) entirely abrogates nuclease activity (Supplementary Figure S2 and (16,35)), although, it is not known which metal ions(s) are used by SNM1B for catalysis *in vivo*, and whether the singly metal ion bound form of SNM1B retains nuclease activity. Nonetheless, when examining the structures of WT SNM1B obtained to date (Figure 1E; Supplementary Figure S8; PDB: 5AHO (24)), it is evident that the first metal ion-binding site (containing four protein ligands) coordinates the metal ion significantly tighter than the second (containing only three protein ligands), which can be lost during purification or crystallisation.

### Features of the active site of SNM1B_1–355_ relating to mechanism and selectivity

The binding of AMP_1 and AMP_2 informs on the mechanism of SNM1B since their positioning and coordination network likely resembles the product of the exonucleolytic cleavage reaction. The structure obtained in this study, WT_1–355_ (nucleotide form), and the geometry of the active site, support the outlined mechanism proposed for SNM1B (24,35), which is analogous to that suggested for other MBL/β-CASP family members, including RNase J (Figure 3B) (38).

Additionally, a role for H276 (motif B, Figure 1E) and D145 (motif A, Figure 1E) in acid base catalysis is likely. This motif is reminiscent of a ‘catalytic dyad’ with the histidine of H276 hydrogen bonding to the 5′ oxygen of AMP_2 opposite from expected site of nucleophilic attack and likely functions a general acid to protonate the oxyanion in the reaction product, as has been suggested for related enzymes (26). In support of this hypothesis, an H276A variant form of SNM1B_1–355_ shows negligible digestion of a ssDNA substrate in a gel-based nuclease assay and exhibited no discernible nuclease activity in a real-time fluorescence-based nuclease assay (Figure 3D & Supplementary Figure S11). This variant also shows a significant reduction in DNA binding (Figure 3C), indicating an important role in that process.

To explore features of the active site relating to the preference of SNM1B for DNA over RNA, we made the S34A substitution (Supplementary Figure S1), because the S34 hydroxyl is located adjacent (∼3 Å) to the second carbon of the ribose ring of AMP_1, which may cause a steric clash with an RNA substrate (Figure 1E). However, mutation of S34 markedly reduced DNA binding, and completely abrogates SNM1B_1–355_ activity (Figure 3C, D and Supplementary Figure S11). It is possible that S34 is involved in polar interactions with the phosphate of the next nucleotide in the DNA substrate. Interestingly, S34 is conserved in human SNM1A and yeast Pso2p where it may make similar contacts, but not in human SNM1C/Artemis, where the DNA binding architecture of the active site differs, reflecting its endonucleolytic, rather than exonucleolytic role.

### There are no observable structural or biochemical differences between WT SNM1B_1–355_ and the breast cancer associated variant, H61Y, SNM1B_1–355_

An SNM1B coding variant, H61Y, correlates with an increased risk of breast cancer (29). This substitution is in the MBL domain, being located adjacent to the active site (24). The effect of this variant on the DNA binding and nuclease activity of SNM1B has yet to be elucidated, and therefore, we purified truncated recombinant, H61Y SNM1B_1–355_. WT and H61Y SNM1B_1–355_ showed similar thermal denaturation profiles, suggesting that the H61Y variant does not substantially perturb its fold (Supplementary Figure S12). H61Y SNM1B_1–355_ exhibits comparable nuclease activity to WT SNM1B_1–355_ in a gel-based activity with simple ssDNA substrates (Supplementary Figure S13A), and more complex substrates (Supplementary Figure S13B), including those containing DNA damage relevant modifications (Supplementary Figure S13C). The H61Y Michaelis-Menten kinetics, as measured by a real-time fluorescence-based nuclease activity were comparable to WT (Supplementary Figure S14A and B), as was its ability to bind ssDNA in the FP binding assay (Supplementary Figure S14C). We solved a structure of H61Y SNM1B_1–355_ (3.2 Å resolution) (Supplementary Figure S15). The overall architecture of this is extremely similar to WT with a backbone root mean squared deviation (RMSD) of 0.3 Å (312 residues to 312 residues). The structure around the active site is also near identical. There is only minor perturbation of the Y61 neighbouring residues (R60, Q63, R206, R207 and E209), and no effect to the α-helix that Y61 resides in, or the adjacent helix, which contains many of the nearby residues (Supplementary Figure S15).

### The putative DNA binding groove contributes to the extended active site of SNM1B

**Figure 5.**
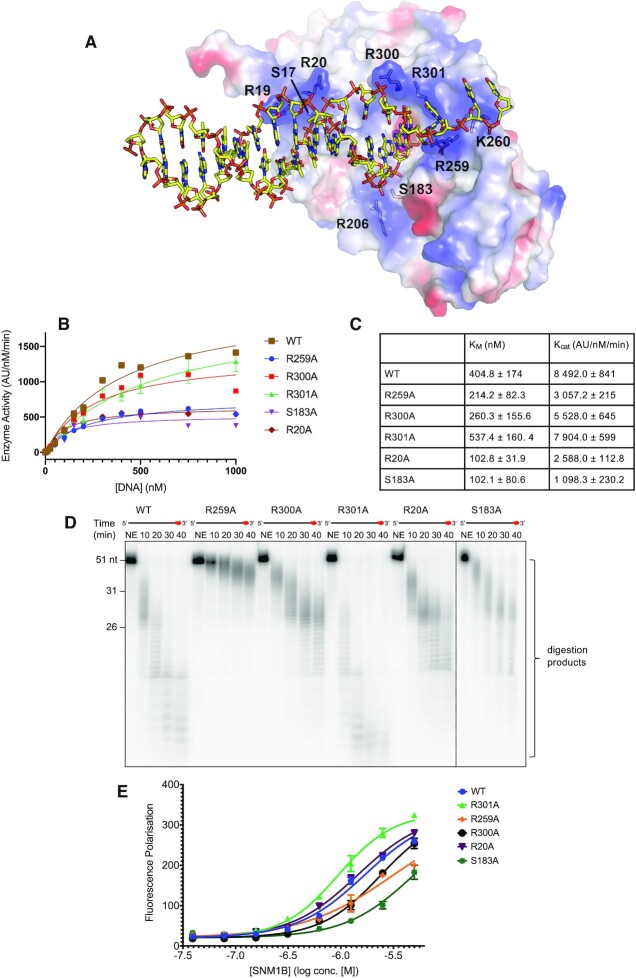
Understanding the extended active site of SNM1B_1–355_. (**A**) Model showing a duplex DNA substrate interacting with the positively charged DNA binding cleft of SNM1B_1–355_. The surface is coloured by electrostatic potential – blue: more positively charged regions, and red: negatively charged. Residues contributing to the assigned DNA binding cleft are as indicated. (**B**) Michaelis-Menten kinetics for WT and phosphate binding pocket mutant SNM1B_1–355_ proteins. Data were obtained *via* real-time fluorescence-based nuclease assay, with at least three experimental repeats for each. 0.25 nM SNM1B_1–355_ was incubated with increasing amounts of DNA substrate (between 1.0 and 1000 nM) and fluorescence readings were taken every 150 sec for 35 min. Error bars: SEM (*n* = 4). (**C**) Table showing the *K*_M_ and *k*_cat_ values for WT and phosphate binding pocket mutants of SNM1B_1–355_. Values determined by fitting Michaelis–Menten curves using Graphpad-Prism software. The ±error values indicate the 95% confidence interval. (**D**) Specific residues within the putative DNA binding cleft contribute to SNM1B_1–355_’s nuclease activity. Substitutions within this region have varying effects on nuclease activity. 1.0 nM SNM1B_1–355_ was incubated with 10 nM ssDNA at 37°C for the indicated time period. Reactions were subsequently analysed by 20% denaturing PAGE. A red asterisk indicates the 3′ radiolabel on the ssDNA substrate. The size of oligonucleotide markers is indicated on the left-hand side of the gel (length in nucleotides). Gel shown is representative of at least three individual experiments, which show qualitatively comparable results. (**E**) Mutations to residues within the putative DNA binding have variable effects on DNA binding capacity compared with WT SNM1B_1–355_ on a 3′ overhang duplex DNA substrate. DNA binding determined by a fluorescence polarisation DNA binding assay for WT and mutant SNM1B_1–355_ proteins. 50 nM 3′ overhang DNA substrate containing a 3′ fluorescein (Supplementary Table S1), was incubated with serially diluted SNM1B_1–355_ (concentrations as indicated) for five min. at room temperature, before fluorescence polarisation was measured. Error bars indicate the SD (*n* = 4). However, the same note should be made, as for Figure 2F, that the binding curves do not saturate, and thus absolute K_D_ values should be interpreted cautiously. Nevertheless, there is a clear, observable reduction in binding affinity for some mutants (R300A, R259A), as seen in the DNA binding curves.

We used the SNM1B_1–355_ (nucleotide form) structure as a guide for modelling SNM1B**_1–355_** complexed with a relevant substrate. Across the front surface of SNM1B, and adjacent to the active site, is a prominent cluster of positively charged residues, with appropriate dimensions for a DNA binding groove. Utilising the orientation of the two active site bound AMP molecules, it was possible to model a B-form duplex DNA substrate. This substrate contained a short 3′-overhang, to reflect the predominant physiologically relevant (telomeric) SNM1B substrate (Figure 5A). There are a number of residues along this putative DNA binding cleft that contribute to the positively charged/polar surface, i.e. S17, R19 and R20, all located between β-strands two and three of the MBL domain; R259 and K260, located in a loop between two parallel β-strands of the β-CASP domain; S183, located in a loop between a β-strand and an α-helix in the β-CASP domain; and R300 and R301, located in a loop region after β11 in the MBL domain and prior to the C-terminal α-helix at the C-terminus).

The model maintains standard Watson-Crick hydrogen bonding up to the point of the 5′-terminal nucleotide, which is partially disrupted (only one hydrogen bond remains, Figure 5A). In the model the base of the terminal nucleotide is twisted with respect to its partner and is stabilised by the aforementioned interactions with R259 and R301 (Figure 5A). The local active site architecture of SNM1B likely only permits one strand of duplex DNA to enter the active site and as this strand is exonucleolytically processed, a 3′-overhang is generated. Interestingly, this region of positive charge across the face of SNM1B extends slightly beyond the catalytic core; and R259 and K260 provide a positively charged surface that may be important for binding to the phosphodiester backbone of the newly generated ssDNA overhang (Figure 5A).

To analyse the contributions of residues within this region to the DNA binding and nuclease activity of WT SNM1B_1–355_, we performed alanine substitutions, the effect of which were variable: R301A has no effect on nuclease activity, kinetics, or DNA binding; all other mutations had negative effects, albeit some more pronounced than others (Figure 5B–E). The effect of S183A in the gel-based nuclease assay was the same as that reported (24), and comparable to those of R300A, and R20A. The effect of R259A was more pronounced. In the real-time fluorescence-based nuclease assay R301A and R300A, showed near WT *K*_M_ and *k*_cat_ values, whereas S183A, R20A and R259A all exhibited decreased *K*_M_ and *k*_cat_ values (Figure 5B and C). With the FP binding assay R301A and R20A were similar to WT SNM1B (R301A was slightly enhanced, with a *K*_D_^app^ of 0.94 μM), whilst R300A and R259A exhibited an increased *K*_D_^app^, and S183A exhibited the largest increase of around 3-fold (Figure 5E). The results of the DNA binding assay are in general agreement with the turnover results.

One observation made from the WT (nucleotide form) structure and from the model of duplex DNA with SNM1B_1–355_, is that there is no apparent mechanism to promote release of the hydrolysed substrate from the active site, whilst SNM1B remains bound to the DNA. This suggests that the DNA substrate that is being processed must be released after each exonucleolytic event to allow the 5′-terminal nucleotide to vacate the active site. This proposal may be reflected in the observation that SNM1B exhibits low processivity, with the exonucleolytic digestion of a DNA substrate being partially inhibited by the addition of 1.0 μM unlabelled ssDNA (a ten-fold molar excess), and completely inhibited by the addition of 10 μM unlabelled substrate (a 100-fold molar excess) (Supplementary Figure S16).

### The open nature of the active site likely confers the ability of SNM1B to digest past DNA damage lesions

**Figure 6.**
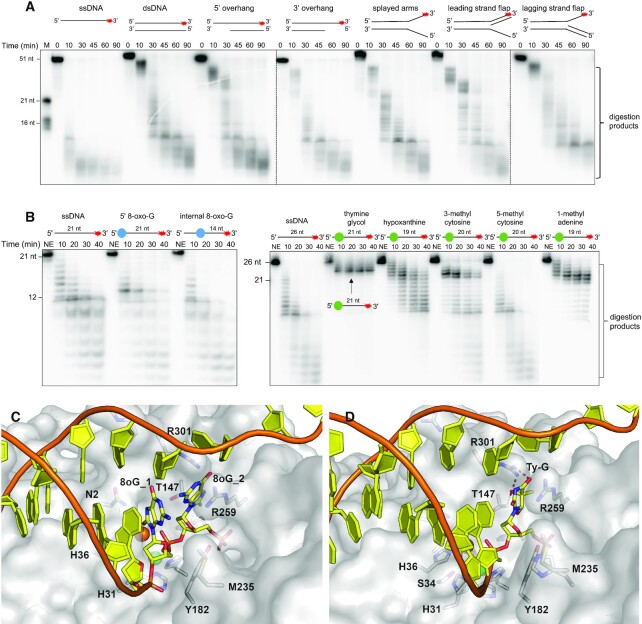
The extended active site of SNM1B_1–355_ may confer a broad range of substrate acceptability and an ability to digest past certain DNA damage lesions. (**A**) SNM1B_1–355_ exhibits efficient exonucleolytic digestion of a variety of structurally diverse DNA substrates. 1.0 nM SNM1B_1–355_ was incubated with 100 nM ssDNA at 37°C for the indicated time period. (**B**) SNM1B_1–355_ digests past different DNA damage lesions, except for a thymine glycol. 1.0 nM SNM1B_1–355_ was incubated with 10 nM ssDNA (either undamaged, or containing a DNA damage lesion where indicated) at 37°C for the indicated time period. The blue circle indicates the location of the 8-oxo-guanine base (either 5′ or internal), and the green circle indicates the location of other damaged nucleotides. In the case of the thymine glycol containing DNA substrate, the point at which SNM1B stalls is indicated. (**C**) A model of duplex DNA substrate containing two 8-oxo-guanines is well accommodated in the active site of SNM1B_1–355_. The SNM1B_1–355_ (nucleotide bound) structure is shown in surface view, with the duplex DNA modelled in dark yellow, and the two terminal 8-oxo-G modified bases labelled (8oG_1 and 8oG_2). Sidechains of residues in SNM1B_1–355_ which are proximal and/or contribute to substrate binding are labelled. (**D**) A thymine glycol modelled lesion (Ty-G) is not well accommodated in the SNM1B_1–355_ active site making steric clashes with R301 and T147 side chains, due to a fairly narrow constriction around the nucleobase. The surface rendering, duplex DNA, and residue side chains are shown and labelled as in (C). Steric clashes with the Ty-G are depicted with red dashes. For all nuclease assays, reactions were analysed by 20% denaturing PAGE; markers are indicated in ssDNA nucleotide length. The nucleotide annotations on damaged substrates indicates the nucleotide length from the DNA lesion to the 3′ end. The secondary structure of substrates is as indicated and a red asterisk indicates the position of the 3′ radiolabel. All gels shown are representative of at least three individual experiments, which show qualitatively comparable results.

Like SNM1A, SNM1B exhibits robust nuclease activity on a diverse range of DNA substrates (Figure 6A) and has been shown to digest past a site-specific ICL *in vitro* (albeit with reduced efficiency relative to SNM1A) (24), and SNM1B-depleted cells are sensitive to IR (9). We examined the ability of SNM1B to digest past common DNA damage lesions, including those that result from oxidation (8-oxo-G, thymine glycol), deamination (hypoxanthine), and methylation (3-methyl cytosine, 5-methyl cytosine, 1-methyl adenine). SNM1B_1–355_ can efficiently digest past a 5-methyl cytosine, and 8-oxo-G; exhibited decreased, but complete, digestion past 3-methyl cytosine and hypoxanthine; and can digest past 1-methyl-adenine, but with markedly reduced efficiency. Interestingly, thymine glycol (a common lesion induced by reactive oxygen species) acted as a complete barrier to digestion. SNM1B was able to digest up to the site of the lesion (i.e. four exonucleolytic events from the 5′ end), but was unable to digest past the thymine glycol leaving a 22 nt substrate with the 5′ end being the thymine glycol modified base (Figure 6B). Thymine glycol is a poorly mutagenic lesion but does act as a barrier to both replicative and repair DNA polymerases (40–42).

We assume that in order to digest fully past a lesion modified bases must be accommodated in both the hydrolysis and 5′-phosphate position, and we have used our SNM1B DNA binding model to try and understand the structural basis of this specificity. The general exonucleolytic promiscuity appears to be the result of the open nature of the active site, with modifications which are planar to the nucleobase (such as the common 8-oxo-G), being well-tolerated in both the hydrolysis and 5′ phosphate position (Figure 6C). In contrast, the thymine glycol base modification is non-planar and features additional hydroxide groups at the five and six positions. Modelling the common 5R6S oxidative version of this lesion in the active site of SNM1B suggests that it may well be accommodated in the hydrolysis position but not in the phosphate-binding pocket which is narrower around the region of the nucleobase, with the potential for steric clashes with nearby R301 and T147 (Figure 6D).

## DISCUSSION

SNM1B is central to human genome integrity, likely through its dual contribution to both telomere maintenance and DNA damage tolerance. Understanding the structure-function relationship of SNM1B may provide useful insight as to how it carries out its cellular role(s). Our structure of SNM1B_1–355_ complexed with two AMP molecules in the active site (1.8 Å resolution) provides insight into its mechanism, and substrate recognition. The position of AMP_1 in the extended active site of SNM1B_1–355_ enables definition of a phosphate binding pocket, which likely confers the strict 5′-phosphate requirement for the exonucleolytic activity of SNM1B (and SNM1A). The identification of this phosphate-binding pocket, whilst previously hypothesised (24), had not been identified for, and the formation of this feature may be induced, in part, by the presence of substrate. The residues that constitute the phosphate binding pocket are conserved with SNM1A, but not SNM1C/Artemis, and the structure of SNM1C/Artemis reveals the aromatic sidechain of Phe308 partially occludes this pocket ((27) PDB: 6TT5). Therefore, the presence of a phosphate binding pocket may provide a key functional delineation between the exonucleolytic and endonucleolytic MBL-β-CASP family members.

A mutational analysis of SNM1B_1–355_’s phosphate binding revealed varying detrimental effects on activity. The effects on DNA binding within this region were similarly varied, but less pronounced, with mutations having, at most, moderately negative effects. These results are consistent with the observation that SNM1B_1–355_ will bind (albeit more-weakly) a 5′-hydroxyl-containing DNA substrate, but shows a complete inability to exonucleolytically digest it, compared to a substrate bearing a 5′-phosphate. Nine hydrogen bonds are made within the context of the phosphate-binding pocket and the pocket likely provides an important contribution to DNA binding (Figure 1D); however, given the multiple contacts, which may contribute to robust activity with multiple substrate types, it is likely that the full effect of the loss of this feature is not manifest in the context of single alanine substitutions. Nevertheless, all phosphate-binding pocket substitutions led to reduced activity, whilst the more moderate effects on DNA binding suggest that substrate interaction was occurring in a less productive, or non-productive, manner. Interestingly, our work also reveals the structural basis for the lack of processivity of SNM1B, since no exit tunnel or route is apparent in our structures. The presence of an ‘exit tunnel’ is present in some RNA-processing β-CASP/MBLs (e.g. RNase J (37,38,43)), but has not been identified in any of the DNA-processing enzymes (e.g. SNM1A, SNM1B and SNM1C/Artemis).

From this nucleotide bound structure, we generated a duplex DNA/SNM1B_1–355_ bound model enabling us to probe the contribution of residues within the putative DNA binding cleft to DNA binding and nuclease activity through site-directed mutagenesis. Individual substitutions within this region had varying effects, likely representing the importance of the entirety of this region, and not the sole importance of single residues. Consistent with their importance, however; R19, S183 and R259 are conserved across chicken, fish, mouse, human, chimpanzee, and pig SNM1B. R20 and K260 exhibit conservation of charge (variation to K or R, respectively) across the aforementioned species.

We examined the ability of purified SNM1B_1–355_ to digest past DNA damage lesions and modelled the corresponding lesions in the active site of SNM1B_1–355_, using our DNA bound model. Intriguingly, we observed that SNM1B_1–355_ can accommodate (and exonucleolytically process) planar nucleobase lesions; by contrast, in the case of thymine glycol, where the C5 methyl group protrudes axially from the ring of the pyrimidine, SNM1B_1–355_’s exonuclease activity is ablated. Due to their sequence and structure, telomeres are particularly sensitive to reactive oxygen species (ROS)-mediated strand hydrolysis and base oxidation (44,45). Moreover, repression of the canonical pathways for oxidative DNA damage response and repair (e.g. base excision repair, or single-strand break repair) at telomeres, leads to the non-detection and subsequent persistence of oxidative lesions (46). Likely due to this susceptibility, telomeric chromatin is enriched for antioxidant enzymes (e.g. peroxiredoxin-PRDX1) that are critical for ameliorating the detrimental effects of ROS (47). Nevertheless, it is likely that some persist and react with telomeric DNA, inducing a variety of oxidative lesions, including 8-oxo-G. EXO1 and SNM1B are the two predominant exonucleases at telomeres that contribute to resection (17), however, EXO1 has a relatively restricted active site (48), and cannot digest past an 8-oxo-G containing substrate *in vitro* (49). In cases of oxidative damage (particularly 8-oxo-G which significantly perturbs telomeric structure) SNM1B may be utilised to process DNA lesions, outside of its canonical resection role (degradation of the C-rich strand contributing to t-loop formation), and thus contribute to telomeric integrity. Perhaps hinting at this, studies have shown that SNM1B depleted cells are sensitive to IR (9), which induces ROS and 8-oxo-G (amongst other lesions). This an area worthy of future investigation. Regardless, the removal of oxidative lesions at telomeric regions is critical, as their presence and persistence alters telomeric structure, promotes telomerase activity, and may contribute to oncogenesis (50). The preference of SNM1B for ssDNA is also a relevant observation in the context of its telomeric role. It raises the possibility that SNM1B functions in a collaborative manner with the telomere-associated helicase, such as RTEL1, to effectively unwind, and exonucleolytically process newly replicated double-stranded telomeric regions to generate the single-stranded overhang requisite for t-loop formation. Inherited mutations in RTEL1 result in telomere dysfunction and a Hoyeraal-Hreirdarsson syndrome in affected individuals (51), the same disorder that was diagnosed in a child with a splice variant of SNM1B that prevented appropriate subcellular telomeric localisation (18).

Our study on the structure, and properties, of the breast cancer associated, H61Y variant of SNM1B_1–355_ reveal its fold is very similar to unliganded WT SNM1B_1–355_ and appears equivalent to WT SNM1B_1–355_ with regards to thermal stability, DNA binding, catalytic efficiency, and nuclease activity. The H61Y SNP (*rs11552449*) is associated with cancer risk due to its correlation with alternative splicing of the SNM1B and *PHTF1* transcripts, each also associated with increased cancer risk (30) and (for the SNM1B transcript) to cellular sensitivity towards mitomycin C and IR (52). Thus, it would seem likely that the reported deleterious associations of *rs11552449* and cancer risk may be mediated by the effects of alternative transcript splicing, rather than the non-synonymous substitution in the protein-coding sequence.

Our results also raise the question of whether SNM1B can be catalytically active with a single metal ion-binding site occupied – MBL fold enzymes can utilise either one or two metal ions for catalysis. With regards to this, SNM1B (along with other MBL/β-CASP nucleases) has previously been considered a two metal-ion-dependent nuclease (53), and the structures of other family members, including RNase J1 (38), RNase J (37,43), CPSF-73, and CPSF-100 (26) have been solved with two Zn ions in the active site. However, with respect to the DNA processing MBL/β-CASP enzymes, the structures of both SNM1A and SNM1C/Artemis have been solved with either one metal ion or two metal ions present in the active site (PDBs: 5AHR, 6TT5, 7AF1 and (24)). For each of these enzymes, when only the first metal binding site is occupied, the following is observed: the metal shifts slightly toward the second binding site; H36 (or analogous histidine) contributes to the coordination network; and the sidechain of D35 rotates away from the catalytic core (note that this is with the caveat that not all the structures were solved with full occupancy of the metal ions). Compared with the RNA processing β-CASP/MBLs, the DNA processing MBLs have one fewer histidine coordinating residue at the second metal ion binding site (H418 for CPSF-73). As this residue is not conserved, it has been hypothesised that this feature delineates RNA from DNA processing β-CASP/MBLs and contributes to the more open active site observed in the latter group of MBL fold DNA nucleases (24). The coordination network of the second SNM1B metal ion binding site is completed by an oxygen atom from the phosphate (as is observed in WT (nucleotide form)) or a tartrate (as was the case for WT SNM1B: 5AHO); ligand binding may explain why in the unliganded state, this site is unoccupied, i.e. metal ion less tightly held without the ligands.

In summary, the structure of WT SNM1B_1–355_ in complex with two deoxyribonucleoside (product mimicking) molecules provides insight as to how it recognises 5′-phosphate residues, hydrolyses phosphodiester bonds, and interacts with DNA substrates and is able to digest past a subset of key oxidative (and other DNA damage) lesions, a role that might be important for the maintenance of genome integrity, particularly at telomeres.

## DATA AVAILABILITY

Atomic coordinates and structure factors for the reported crystal structures have been deposited with the Protein Data bank under accession numbers: 7B9B, 7B2X and 7A1F.

## Supplementary Material

gkab692_Supplemental_File
