# Supplementary material for: A phosphate binding pocket is a key determinant of exo- versus endo-nucleolytic activity in the SNM1 nuclease family
Source: Nucleic Acids Res. 2021 Aug 13;49(16):9294–309. doi: 10.1093/nar/gkab692 (PMC8450094; doi:10.1093/nar/gkab692)
Supplement: gkab692_Supplemental_File [file gkab692_supplemental_file.pdf]

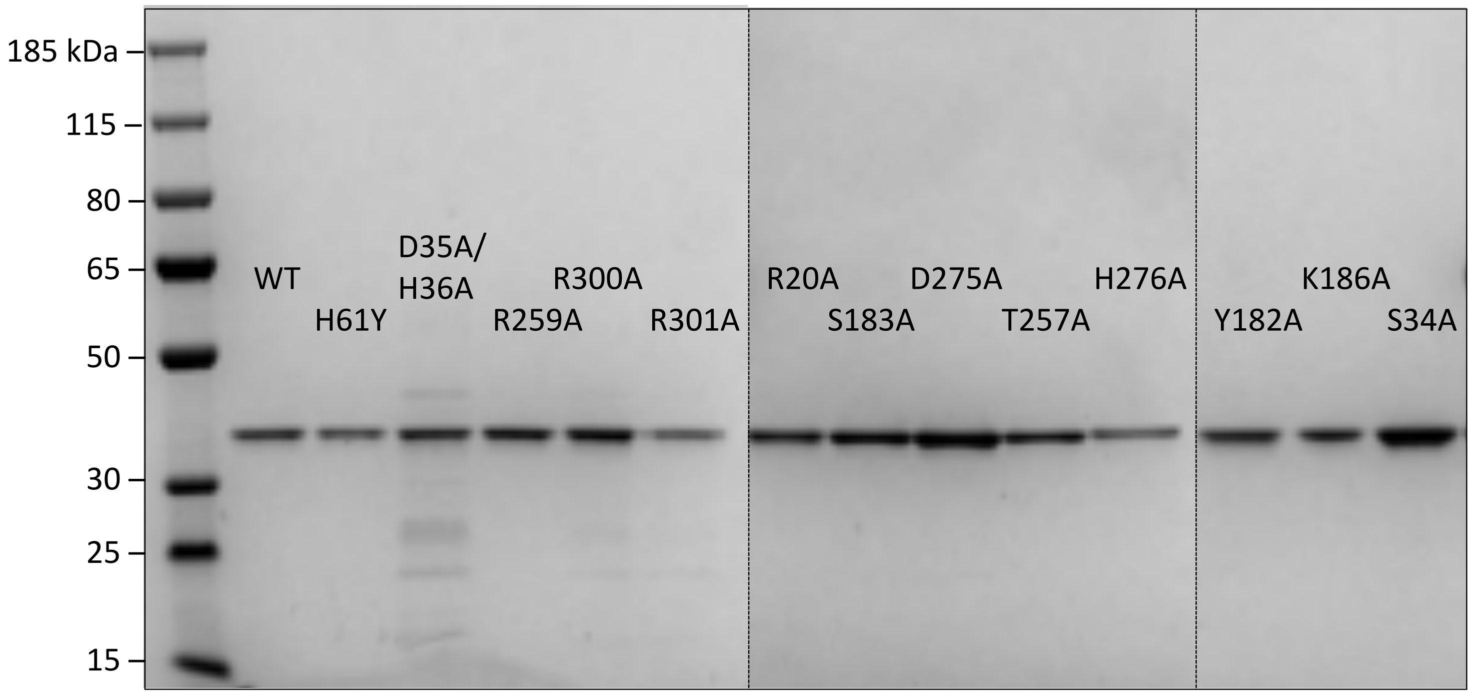

Suppl. Figure 1. SDS-PAGE of purified WT and mutant forms of SNM1B$_{1–355}$.
The purity of WT or mutant SNM1B$_{1–355}$ purified proteins was analysed by SDS-PAGE. The predicted molecular weight for WT SNM1B (1–335) is 38 566.6 Da. Approximate molecular weights based on a molecular weight marker (PageRuler Plus Prestained Protein Ladder; ThermoFisher) are as indicated.
The masses of WT and all mutant SNM1B $_{1–355}$ proteins were validated by ESI-TOF-MS. All SNM1B proteins that were purified with a C-terminal decahistidine-FLAG tag (except D35A/H36A) and were observed to have a +42 Da mass increment relative to that expected. Post-translation modification (PTM) mapping MS identified this being due to acetylation of the N-terminal methionine. D35A/H36A SNM1B$_{1–355}$ (purified with an N-terminal hexahistidine tag), did not exhibit this PTM and the measured mass matched that expected (37 954.9 Da). Protein concentrations were carefully calibrated before use in gel-based and fluorescence-based nuclease assays.

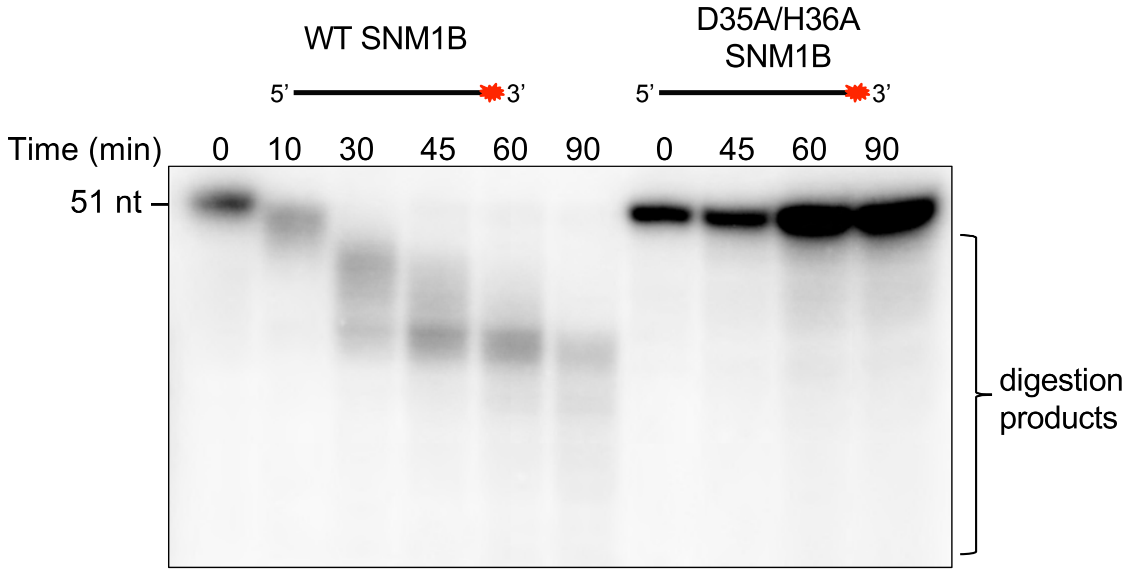

Suppl. Figure 2. Purified D35A/H36A SNM1B$_{1\text{–}355}$ has no discernible nuclease activity on a ssDNA substrate.
The nuclease activity of WT and D35A/H36A SNM1B$_{1\text{–}355}$ was assessed by gel-based assays. 0.75 nM SNM1B$_{1\text{–}355}$ was incubated with 100 nM of ssDNA in a total reaction volume of 10 µL at 37 °C for the indicated time periods; reactions were stopped by addition of 5 µL stop solution with incubation at 95 °C. Products were analysed by 20% denaturing PAGE. Each gel is representative of at least three individual experiments.

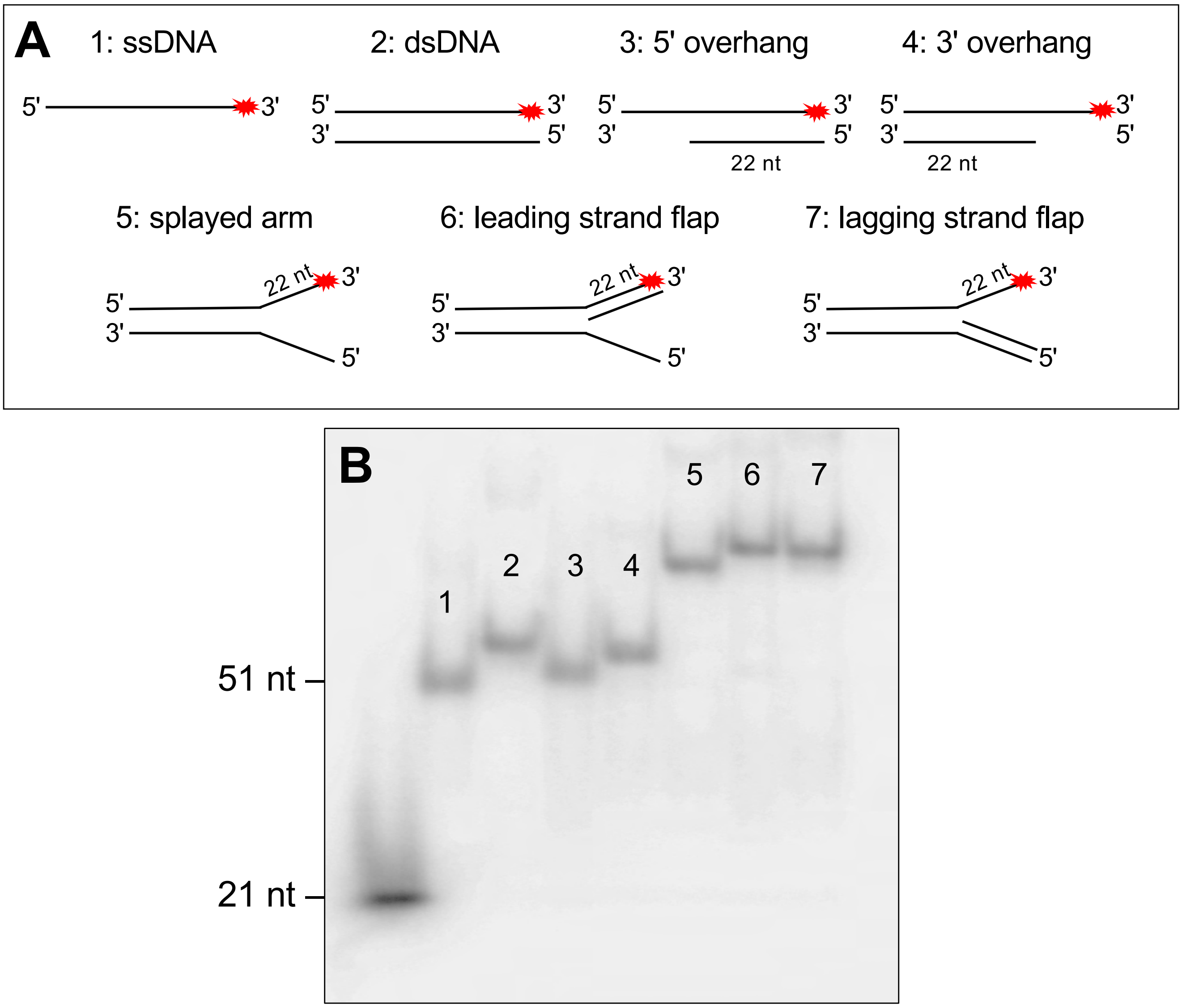

Suppl. Figure 3. Generation of structurally diverse DNA substrates.

A. Single-stranded 51 nt oligonucleotides radiolabelled at the 3' end were annealed with the appropriate corresponding oligonucleotide to generate the substrates shown in schematic form (see Suppl. Table 1 for DNA sequences). Red stars denote the α-^{32}P-dATP label.

B. Non-denaturing PAGE demonstrating correct annealing of oligonucleotide substrates. Labeled as follows: 1 = ssDNA, 2 = dsDNA (annealed strand = 50 nt), 3 = 5' overhang, 4 = 3' overhang, 5 = splayed arm, 6 = leading strand flap, 7 = lagging strand flap.

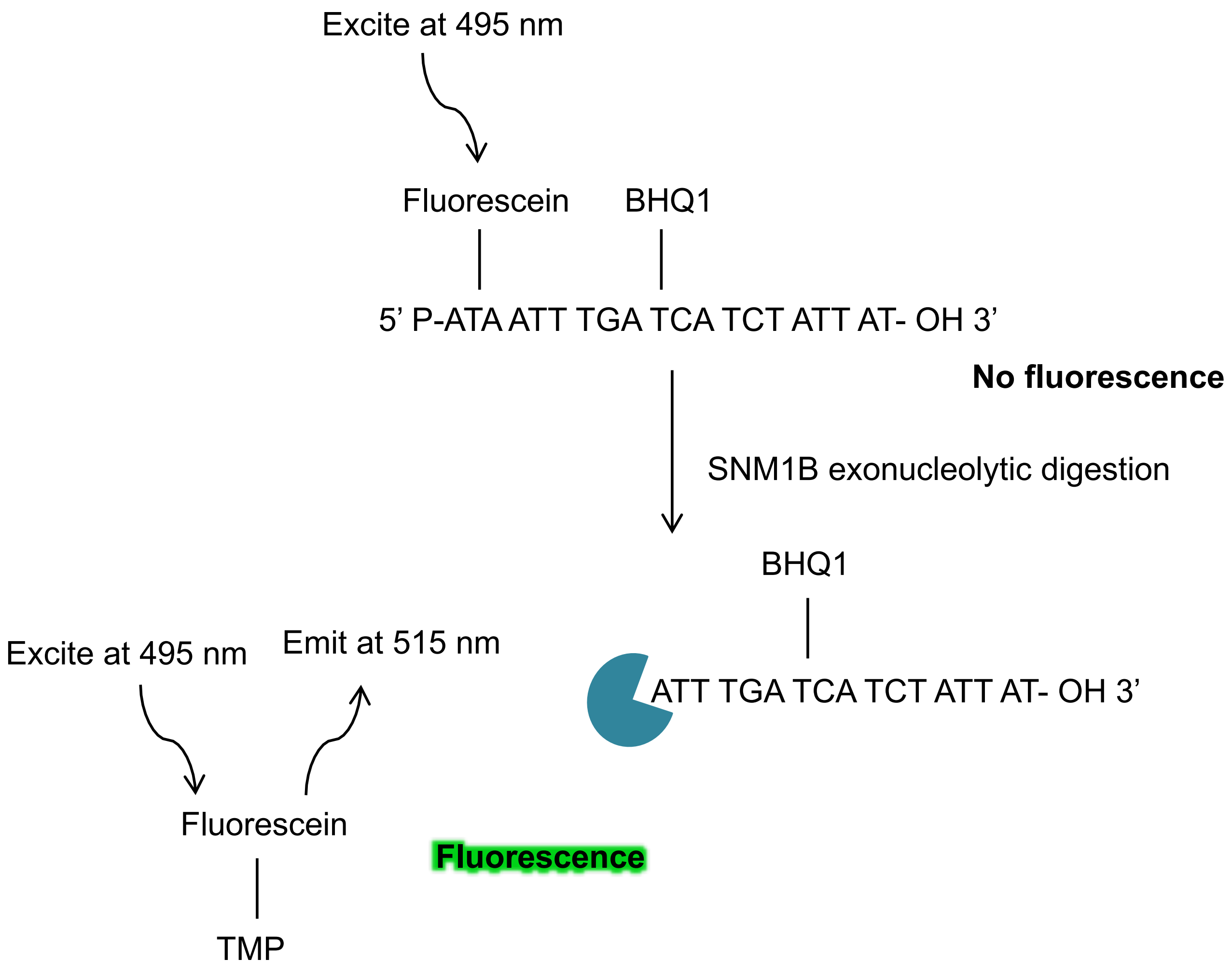

Suppl. Figure 4. Schematic representation of the real-time fluorescence-based nuclease assay.
The intact DNA substrate (Suppl. Table 1) does not fluoresce, due to the proximity of the black-hole-quench (BHQ-1) and fluorescein groups. Following exonucleolytic digestion of SNM1B $_{1-355}$ past the fluorescein-conjugated-T, and subsequent uncoupling of the fluorescein and BHQ-1, there is a concomitant increase in fluorescence that can be measured.

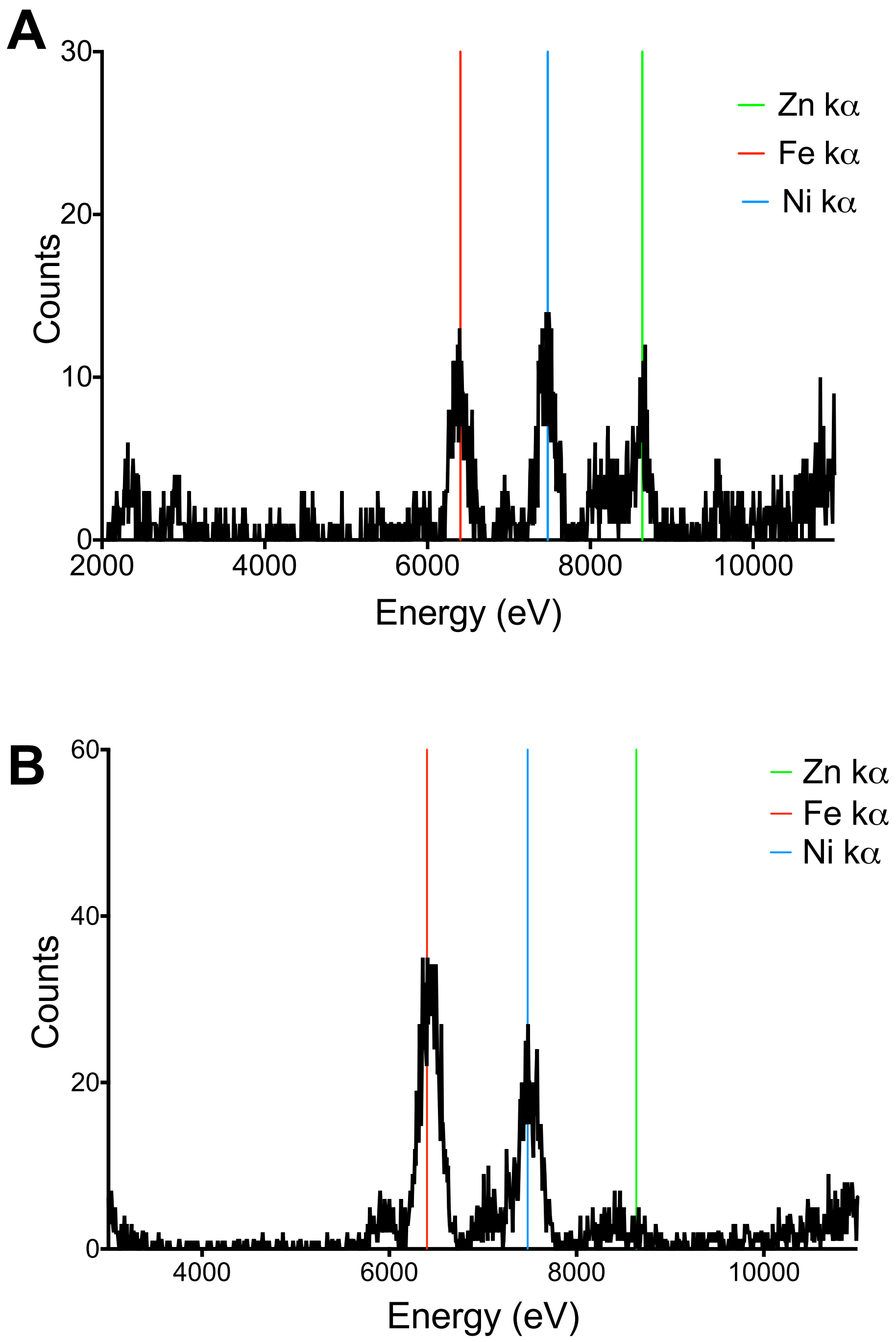

Suppl. Figure 5. X-ray fluorescence of analysis of metals present in the WT SNM1B$_{1-355}$ crystal forms.

A. Zinc, iron, and nickel are present in the SNM1B$_{1-355}$ (apo form) crystal. The k-α emission peaks for each metal are as labelled.

B. Iron and nickel predominate in the SNM1B$_{1-355}$ (nucleotide form) crystal. The k-α emission peaks are as labelled

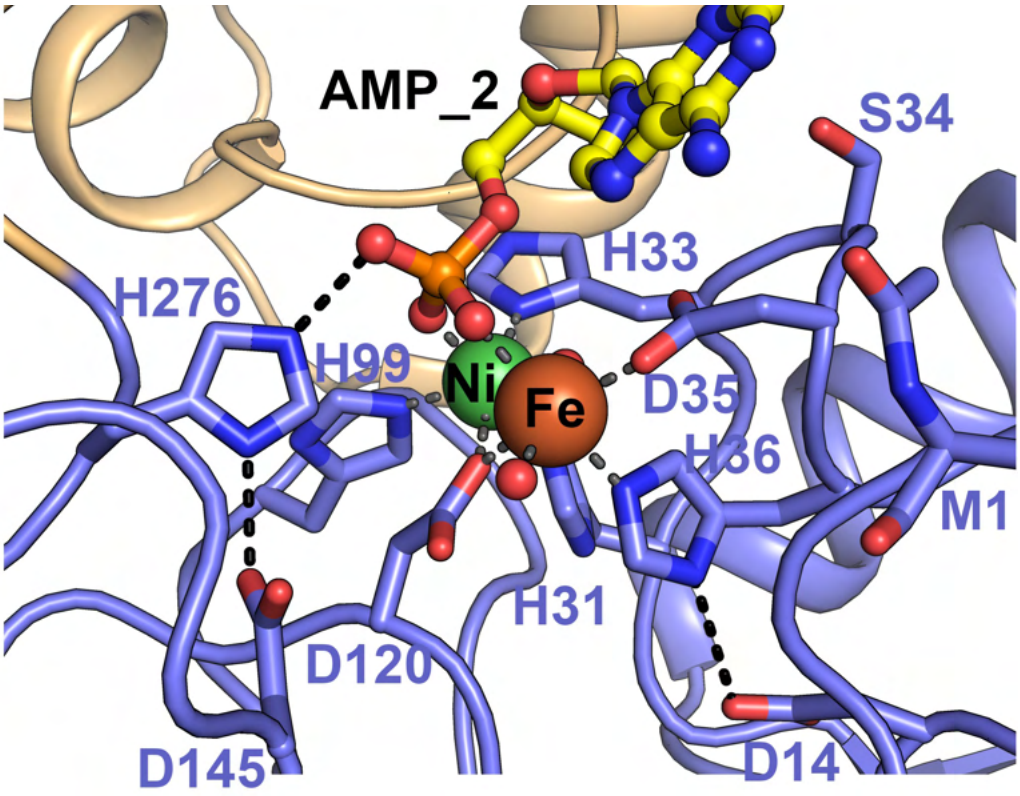

Suppl. Figure 6. The coordination network of the phosphate group of AMP_2 indicates residues likely involved in catalysis and / or binding the product. The active site metal ions (Ni^{2+} at the M1 site, and Fe at the M2 site are as indicated). This is an expansion of Figure 1E.

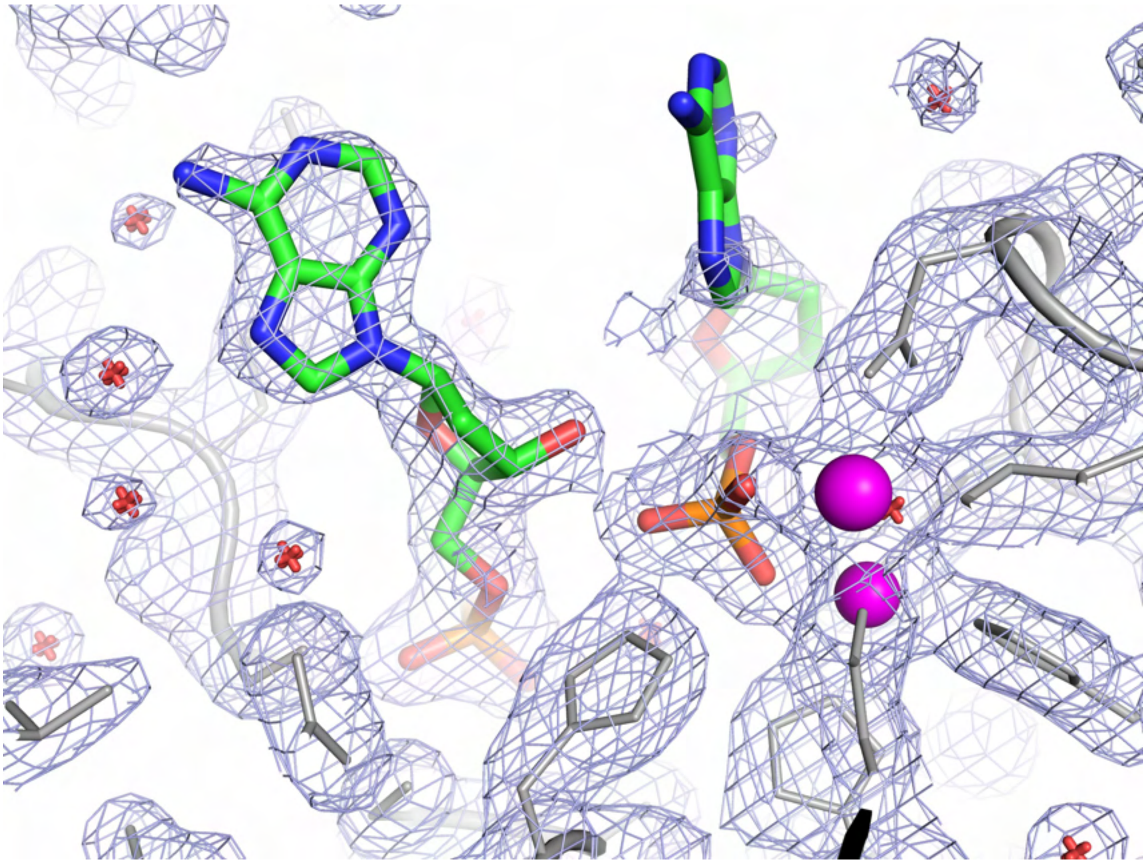

Suppl. Figure 7. Electron density map of the active site of SNM1B$_{1-355}$ (nucleotide form).
A $2F_o$-$1F_c$ electron density map of WT SNM1B $_{1-355}$ nucleotide complex contoured at 1 sigma in the vicinity of the active site, with nucleotides in green and metals in pink.

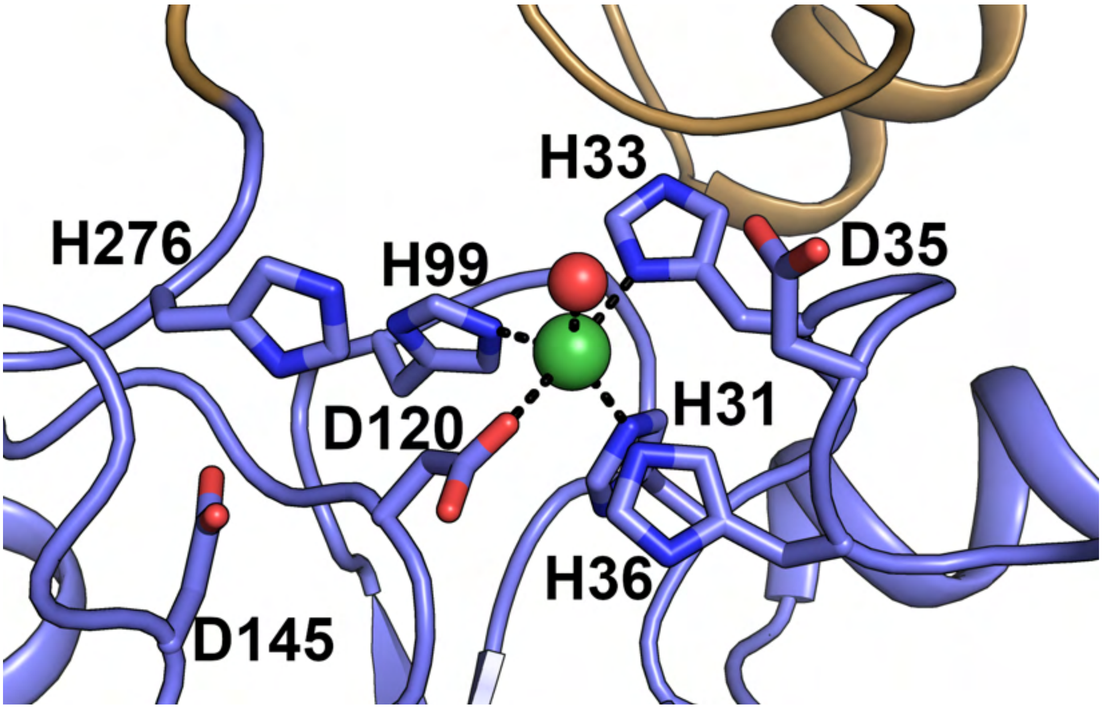

Suppl. Figure 8. Metal ion co-ordination in the active site of SNM1B$_{1–355}$ (apo form).
The unliganded structure of SNM1B$_{1–355}$ (apo form) has only the first metal ion binding site (M1) occupied (modelled as a Ni^{2+} ion). The metal ion coordinating residues are as labelled, the Ni^{2+} ion is depicted as a green sphere, and the coordinating water molecule is a red sphere.

**Cr52** (ng/g)	**Mn55** (ng/g)	**Fe56** (ng/g)	**Co59** (ng/g)	**Ni60** (ng/g)	**Cu65** (ng/g)	**Zn66** (ng/g)
106.063	51.998	799.143	Below DL	741.744	90.883	335.494
167.682	53.296	856.635	Below DL	748.558	95.844	380.470
77.253	42.085	651.868	Below DL	593.975	609.409	295.997

Suppl. Figure 9. Inductively coupled plasma mass spectrometry (ICP-MS) of WT SNM1B $_{1-355}$ (apo form).
ICP-MS data for WT SNM1B $_{1-355}$ (apo form) with the amount of each element detected in nanograms per gram of SNM1B $_{1-355}$ protein. The three rows represent experimental repeats; the third value (lowest row) for Cu65 is probably an outlier.

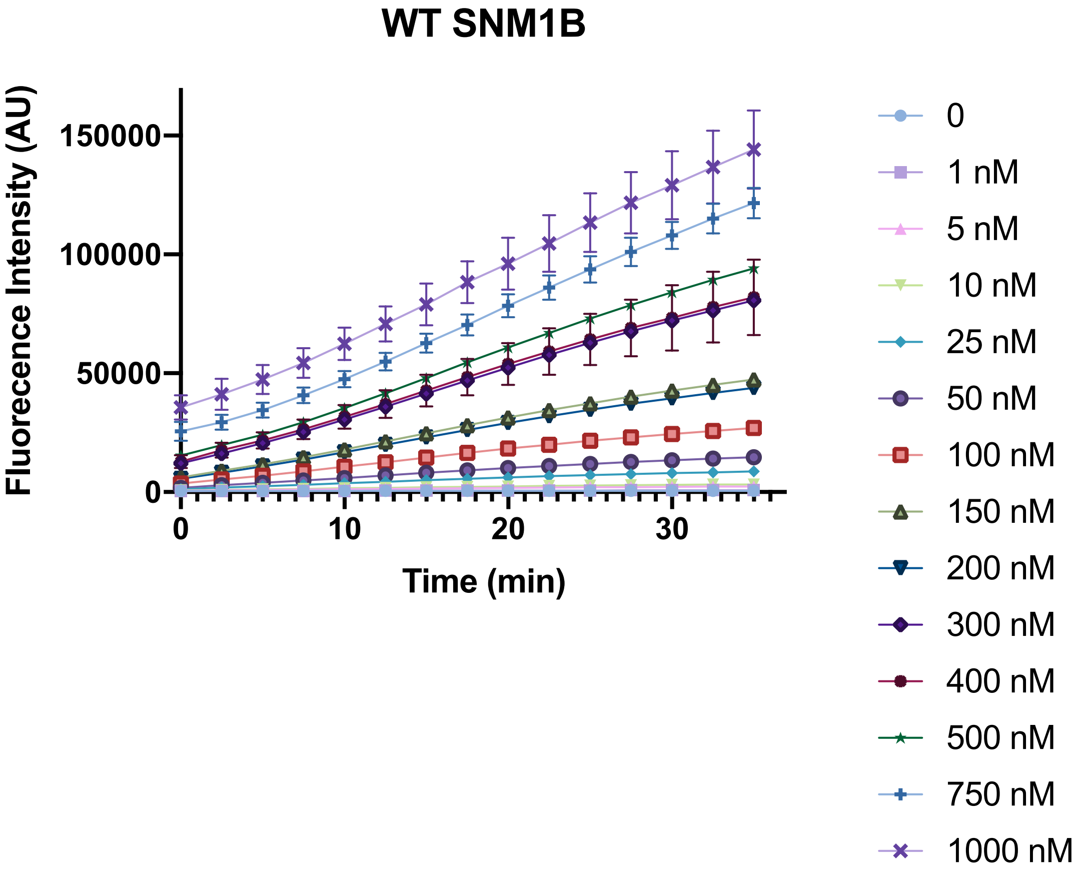

Suppl. Figure 10. Raw data for WT SNM1B $_{1\text{–}355}$ from the real-time fluorescence-based nuclease assay.

Fluorescence intensity plotted against time for WT SNM1B $_{1\text{–}355}$ (0.25 nM) incubated with increasing concentrations (as indicated) of fluorescein-BHQ-1-containing DNA (Suppl. Table 1). The slope of the curve for each DNA concentration was plotted to obtain Michaelis-Menten parameters, as in Figures 2D, 5B, Suppl. 9A, and Suppl. 12A.

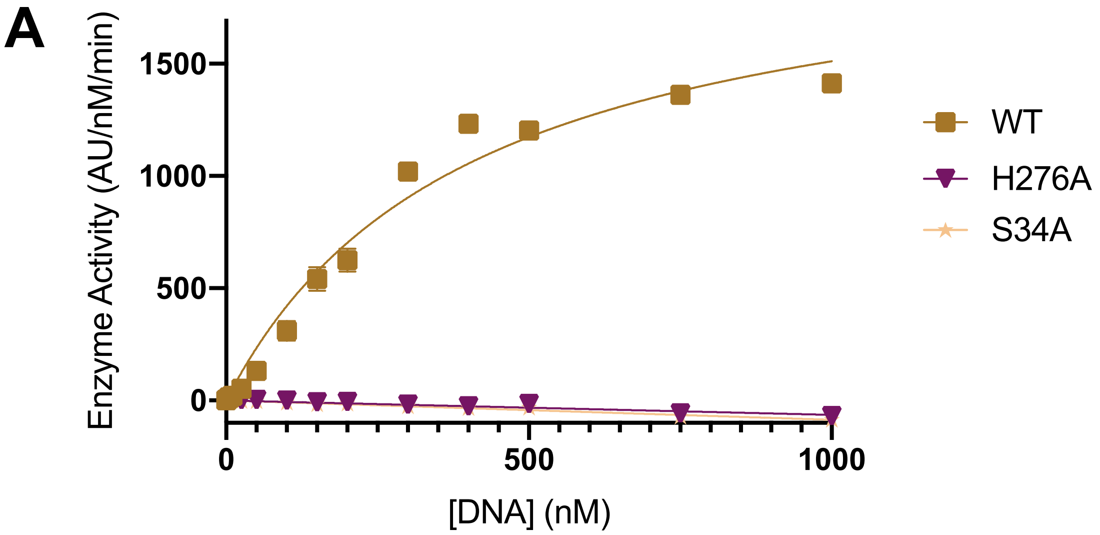

B	K_M (nM)	K_{cat} (AU/nM/min)
WT	405 ± 174	8, 490 ± 841
H276A	unable to be calculated	unable to be calculated
S34A	unable to be calculated	unable to be calculated

Suppl. Figure 11. H276A and S34A point mutations abrogate the nuclease activity of SNM1B $_{1-355}$.

A. Michaelis-Menten curves fitted using the initial velocity of SNM1B in the fluorescence based assay. 0.25 nM of indicated SNM1B $_{1-355}$ and increasing concentrations of ss 21-nucleotide DNA were utilised. Data are from a minimum of three repeats and error bars depict the standard error of the mean.

B. K_M and k_{cat} values for WT or mutant SNM1B $_{1-355}$, fitted using Prism software. Values for H276A and S34A were unable to be calculated due to non-detectable nuclease activity. The ± error values indicate the 95% confidence interval.

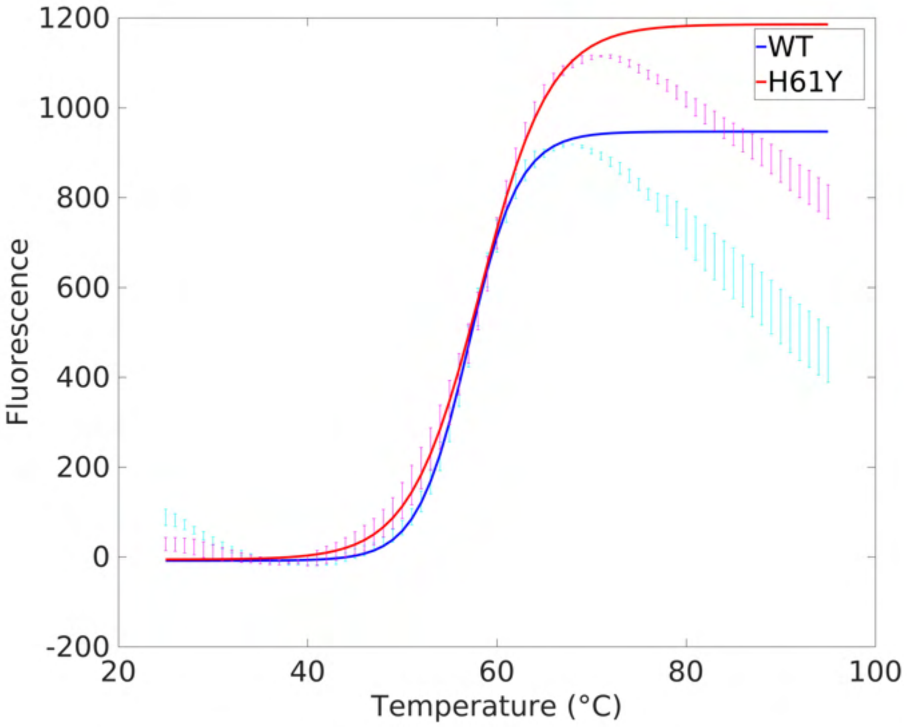

Suppl. Figure 12. The H61Y SNM1B$_{1\text{–}355}$ SNP does not decrease thermal stability compared to WT SNM1B.
Temperature was increased and the fluorescence measured using SYPRO Orange. WT T_m = 57.37°C and H61Y T_m = 57.15°C.

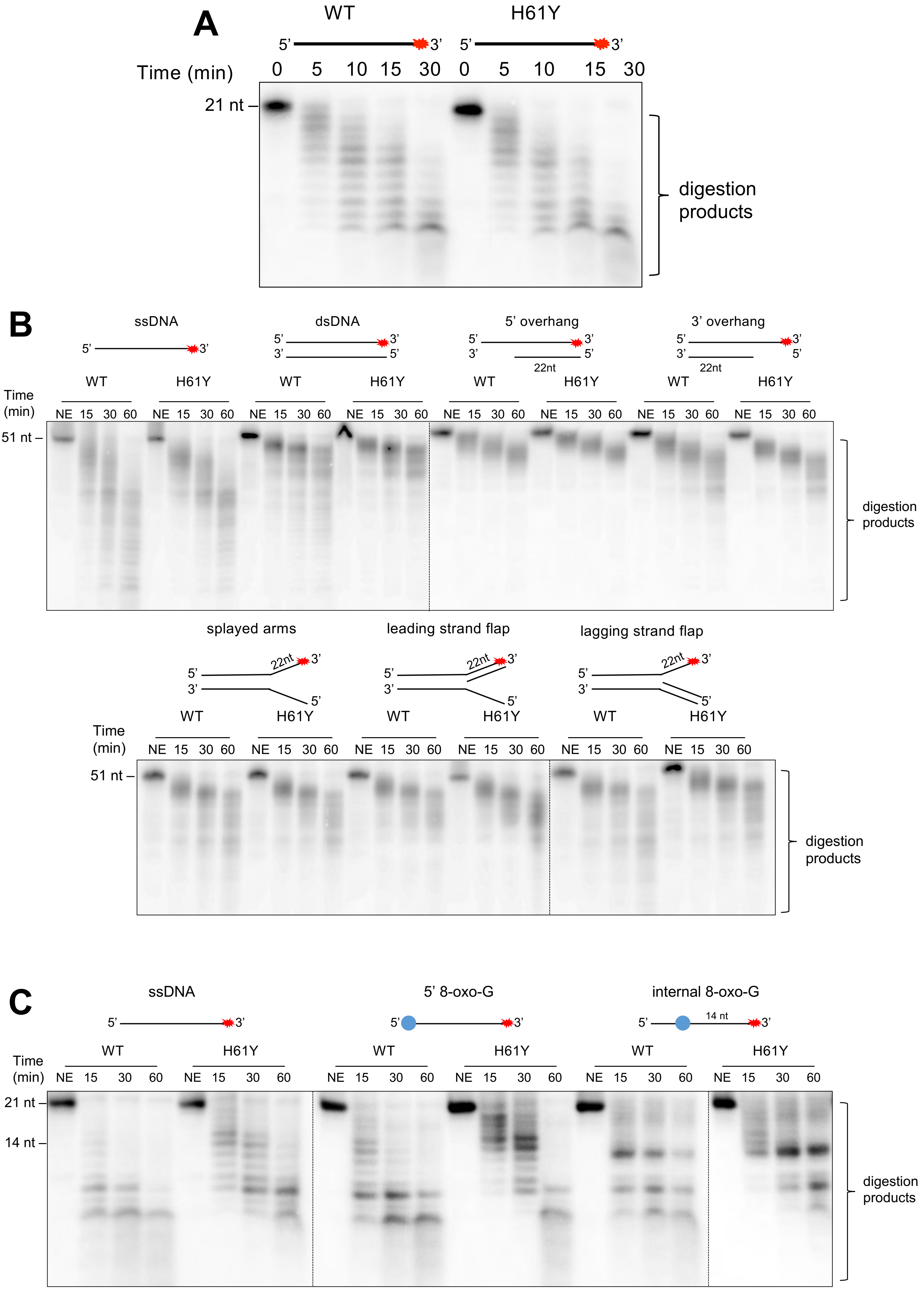
A
WT
H61Y
5'
3'
Time (min)
0 5 10 15 30 0 5 10 15 30
21 nt
digestion products
B
ssDNA
dsDNA
5' overhang
3' overhang
22nt
WT
H61Y
Time (min)
NE 15 30 60
51 nt
digestion products
splayed arms
leading strand flap
lagging strand flap
C
ssDNA
5' 8-oxo-G
internal 8-oxo-G
14 nt
21 nt
14 nt
digestion products

Suppl. Figure 13. WT and H61Y SNM1B $_{1\text{–}355}$ exhibit comparable nuclease activity on simple ssDNA substrates, more structurally diverse DNA substrates, and those that contain DNA damage.

A. With ssDNA the nuclease activities of WT and H61Y SNM1B$_{1\text{–}355}$ are similar. 1.0 nM SNM1B$_{1\text{–}355}$ was incubated with 100 nM 21 nt ssDNA at 37 ^{o}C for the indicated period.
B. Both WT and H61Y SNM1B$_{1\text{–}355}$ exonucleolytically hydrolsye a variety of DNA substrates, with a preference for ssDNA. 100 nM of DNA substrate (as labeled) was added to 1.0 nM SNM1B$_{1\text{–}355}$ and incubated at 37 ^{o}C, for the indicated period.
C. Both WT and H61Y SNM1B$_{1\text{–}355}$ possess the ability to digest past a 5' 8-oxo-guanine, or an internal 8-oxo-guanine base lesion. 100 nM of DNA substrate (either undamaged or containing an 8-oxo-G as indicated) was added to 1.0 nM WT or H61Y SNM1B$_{1\text{–}355}$ and incubated at 37 ^{o}C for increasing times.

Products were analysed by 20% denaturing PAGE. A red asterisk indicates a 3' radiolabel on the ssDNA substrate. The size of oligonucleotide markers is indicated on the left-hand side of the gel (length in nucleotides). Each gel is representative of at least three individual experiments from two separate purifications.

A

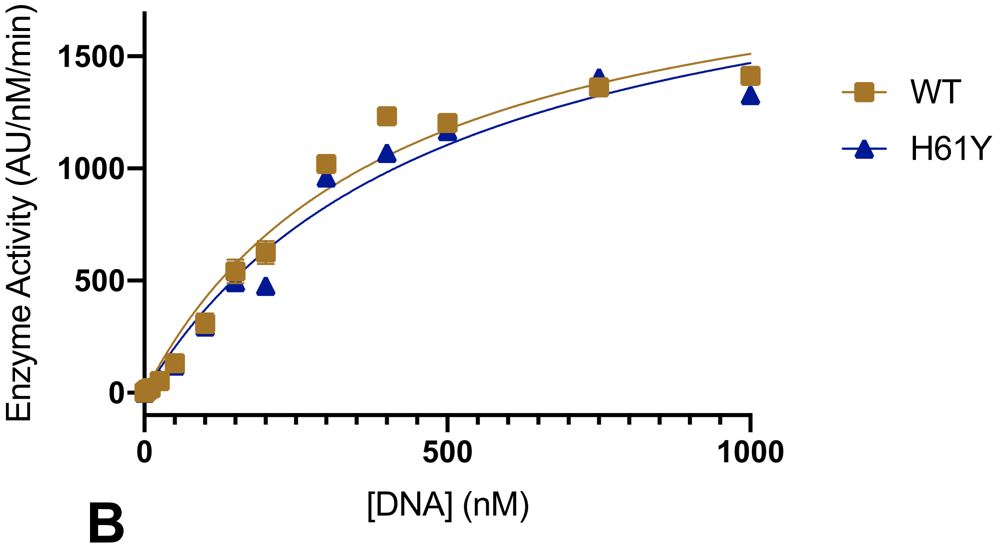

B

	K_M (nM)	K_{cat} (AU/nM/min)
WT	405 ± 174	8, 490 ± 841
H61Y	494 ± 241	8, 780 ± 1 060

C

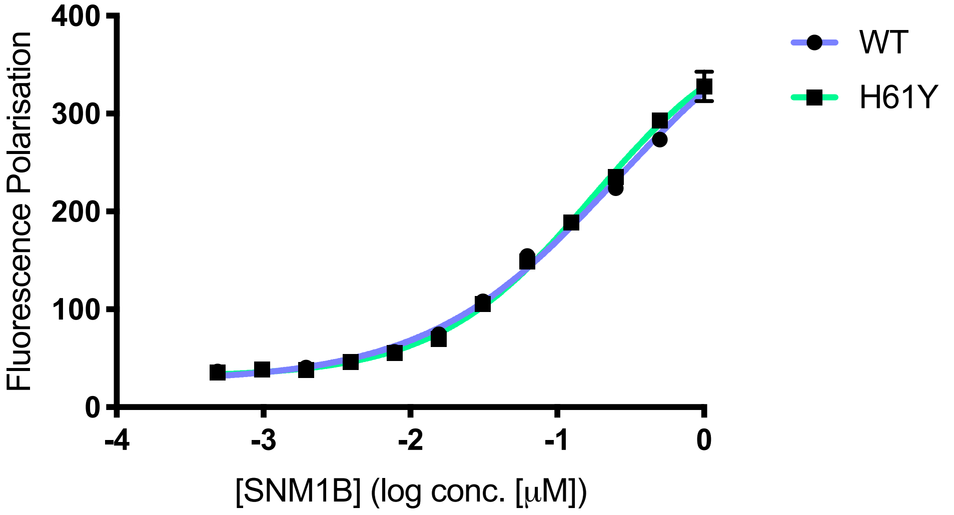

Suppl. Figure 14. Kinetics and DNA binding affinity of WT and H61Y SNM1B $_{1\text{–}355}$ are similar.

A. In a real-time fluorescence-based nuclease assay WT and H61Y SNM1B $_{1\text{–}355}$ exhibit comparable Michaelis-Menten paramters. 0.25 nM SNM1B $_{1\text{–}355}$ was incubated with increasing amounts of DNA (between 1.0 and 1000 nM) and fluorescence readings were taken every 150 sec for 35 min. Error bars indicate the SEM, and at least four experimental repeats were performed.

B. Table showing the K_M and k_{cat} values for WT and H61Y SNM1B $_{1\text{–}355}$. Values were determined by fitting Michaelis-Menten curves using Graphpad-Prism software. The ± error values indicate the 95% confidence interval.

C. WT and H61Y SNM1B $_{1\text{–}355}$ exhibit similar binding affinities with a 20 nt ssDNA substrate. DNA binding was measured by fluorescence anisotropy: 10 nM DNA substrate was incubated with serially diluted concentrations of SNM1B $_{1\text{–}355}$ (as indicated) for five minutes at room temperature. Each reaction was in quadruplicate and at least four experimental repeats were performed. Error bars indicate the SD. WT SNM1B $_{1\text{–}355}$ K_D = 153.2 nM ± 19.64 and H61Y SNM1B $_{1\text{–}355}$ K_D = 175.3 nM ± 15.46.

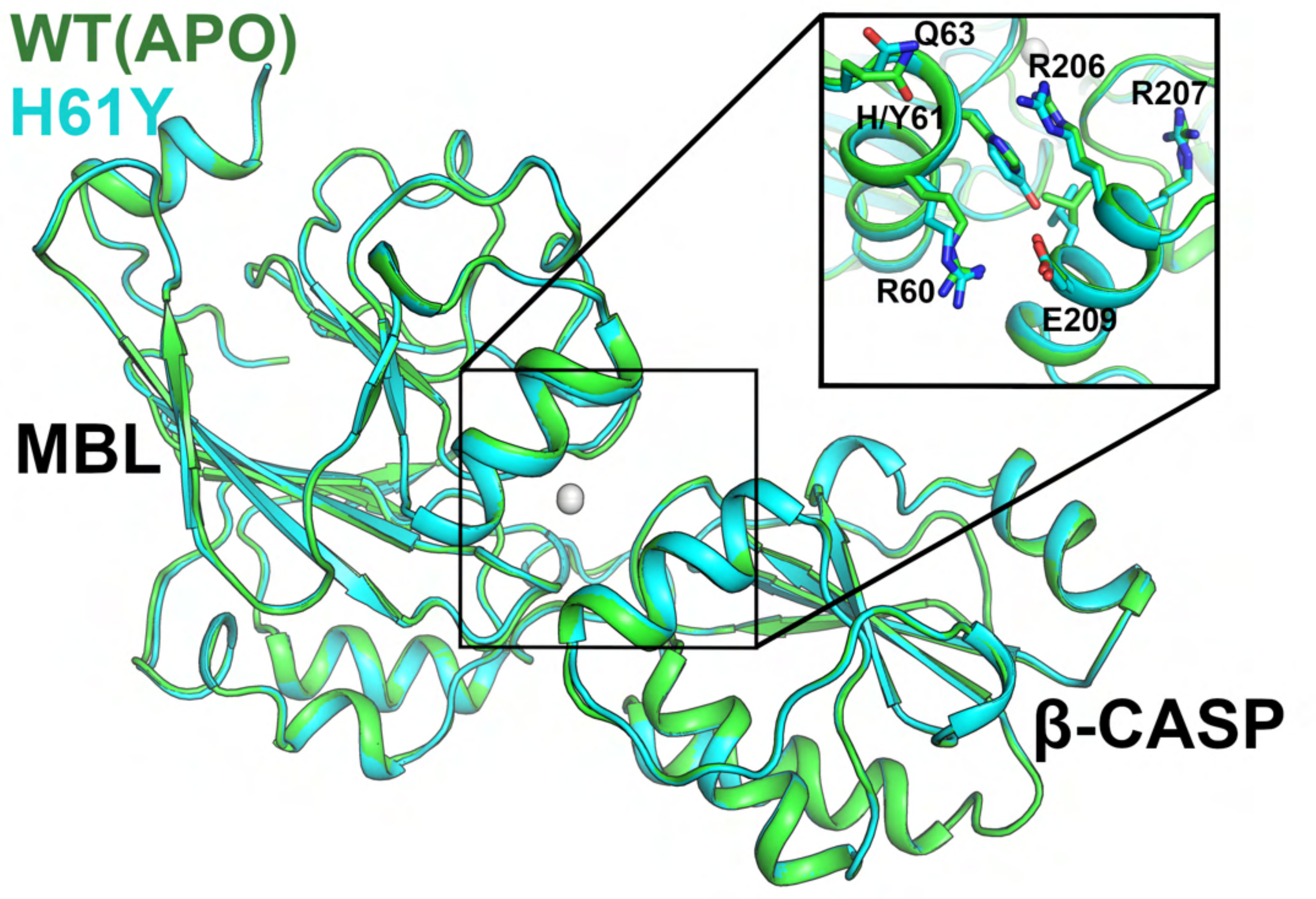

Suppl. Figure 15. The structures of WT (apo form) and H61Y SNM1B $_{1\text{–}355}$ are very similar, both globally, and within the local context of the H/Y61 SNP variant.
Overlay of WT (apo form, solved to 2.8 Å) and H61Y (3.2 Å) SNM1B $_{1\text{–}355}$ represented in cartoon form. The active site metal ion is represented in light grey, and the MBL and β-CASP domains are as labelled. The zoom in panel shows the location and conformation of H/Y61 and neighbouring residues and helices.

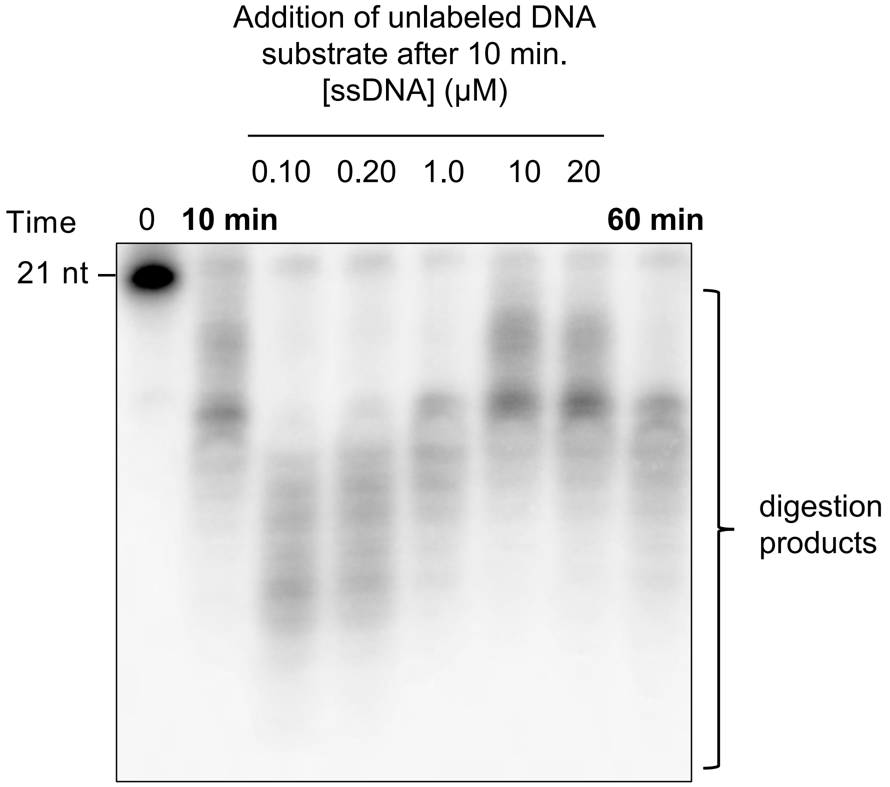

Suppl. Figure 16. The nuclease activity of SNM1B $_{1–355}$ is inhibited by the addition of non-labelled excess ssDNA substrate.

Gel based ‘competition’ assay whereby 100 nM of DNA substrate was added to 1.0 nM SNM1B $_{1–355}$ and incubated at 37 °C for 10 min, at which point the reaction was either stopped, or increasing amounts of unlabeled ssDNA substrate was added to the reaction mix, and then incubated for a total period of 60 min. A single 60 min reaction was included where no additional unlabeled substrate was added. Reaction products were analysed by 20% denaturing PAGE; each gel is representative of at least three individual experiments.

A

Code	DNA oligonucleotide sequence
1	5’ P-ATA AAT ATT TTT TAT TAA TAA TAG ATC ACC TTT CTT TCT CTT CTC CCC TT-OH 3’
2	5’ OH-AAG GGG AGA AGA GAA AGA AAG GTG ATC TAT TAT TAA TAA AAA ATA TTT AT-OH 3’
3	5’ OH-AAG GGG AGA AGA GAA AGA AAG G-OH 3’
4	5’ OH-ATT ATT AAT AAA AAA TAT TTA T -OH 3’
5	5’ OH-TTC CCC TCC TCT CCT TCC TTC CTG ATC TAT TAT TAA TAA AAA ATA TTT AT-OH 3’
6	5’ OH-AAG GGG AGA AGA GAA AGA AAG G-OH 3’
7	5’ P-AT AAT AGA TGA TCA AAT TAT-OH 3’
8	5’ OH-AT AAT AGA TGA TCA AAT TAT-OH 3’
9	5’ P-ATA ATT TGA TCA TCT ATT ATA- OH 3’
10	5’ P-ATA ATT T[8oxoG]A TCA TCT ATT ATA-OH 3’
11	5’ P-[8oxoG]TA ATT TGA TCA TCT ATT ATA-OH 3’
12	5' P-ATA ATC ATG ATC ATC TAT TAT GCG T-OH 3’
13	5' P-ATA A[thymine glycol]C ATG ATC ATC TAT TAT GCG T-OH 3’
14	5' P-ATA ATC [hypoxanthine]TG ATC ATC TAT TAT GCG T-OH 3’
15	5' P-ATA AT[3-methyl cytosine] ATG ATC ATC TAT TAT GCG T-OH 3’
16	5' P-ATA AT[5-methyl cytosine] ATG ATC ATC TAT TAT GCG T-OH 3’
17	5' P-ATA ATC [1-methyl adenine]TG ATC ATC TAT TAT GCG T-OH 3’
18	5' P- A[Fluorescein-T]A ATT TGA [BHQ1-T]CA TCT ATT AT -OH 3'
19	5' P- ATA ATT TGA TCA [Fluorescein-T]CT ATT ATA -OH 3'
20	5’ P-ATA AAT ATT TTT TAT TAA TAA TAG ATC ACC TTT CTT TCT CTT CTC CCC TT-OH-[FITC] 3’

B

3′ labelled substrates (α-^{32}P-dATP)			
**Annealed DNA Sequences**	**Substrate Structure**	**Description**	**Figure(s)**
1*	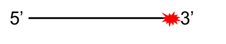	Single-stranded 51 nt DNA with 5’ phosphate	2, 3, 5, 6, Suppl. 2, Suppl. 3, Suppl. 13
1* + 2	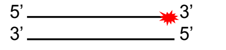	dsDNA	6, Suppl. 3, Suppl. 13
1* + 3	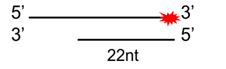	5’ overhang	6, Suppl.3, Suppl. 13
1* + 4	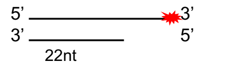	3’ overhang	6, Suppl. 3, Suppl. 13
1* + 5	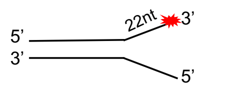	Splayed arm	6, Suppl. 3, Suppl. 13
1* + 5 + 3	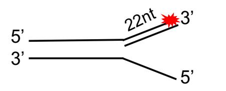	Leading strand flap	6, Suppl. 3, Suppl. 13
1* + 5 + 6	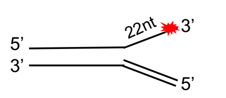	Lagging strand flap	6, Suppl. 13
7*	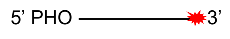	Single-stranded 21 nt DNA with a 5’ phosphate	2, Suppl. 13, Suppl. 16
8*	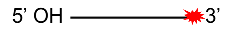	Single-stranded 21 nt DNA with a 5’ hydroxyl	2
9*	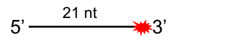	8-oxo-G control substrate (21 nt ssDNA)	6, Suppl. 13
10*	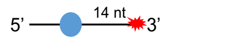	21 nt ssDNA with an internal 8-oxo-G	6, Suppl. 13
11*	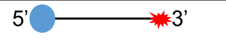	21 nt ssDNA with a 5’ 8-oxo-G	6, Suppl. 13
12*	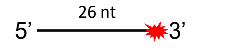	26 nt ssDNA (control oligo for damaged substrates)	6
13*	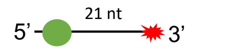	26 nt ssDNA with thymine glycol	6
14*	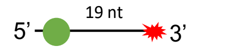	26 nt ssDNA with hypoxanthine	6
15*	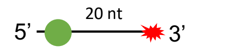	26 nt ssDNA with 3-methyl cytosine	6
16*	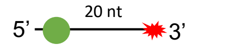	26 nt ssDNA with 5-methyl cytosine	6
17*	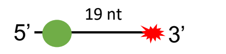	26 nt ssDNA with 1-methyl adenine	6
**Fluorescently labelled substrates**			
18	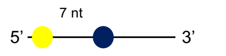	20 nt ssDNA with an internal fluorescein and BHQ-1	2, 5, Suppl. 4, Suppl. 10, Suppl. 11, Suppl. 14
19		20 nt ssDNA with an internal fluorescein	Suppl. 14
20* + 4	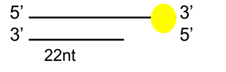	3’ overhang with the 50 nt strand labelled with a 3’ FITC	2, 3, 5

Suppl. Table 1. Oligonucleotide sequences and substrates structures used.

A. DNA oligonucleotide sequences and codes used to denote them. P = phosphate; OH = hydroxyl.

B. The oligonucleotide sequences that are annealed to generate the indicated substrate structures. In the first column, the asterisk indicates the radiolabelled strand. In the ‘substrate structure’ column the red asterisk indicates the radiolabel; a blue circle an 8-oxo-guanine; a green circle, other DNA damage lesions; a yellow circle a fluorescein; and a dark blue circle, a BHQ.
